# Genetic determinants of testicular sperm extraction outcomes: insights from a large multicentre study of men with non-obstructive azoospermia

**DOI:** 10.1093/hropen/hoaf049

**Published:** 2025-08-29

**Authors:** Antoni Riera-Escamilla, Mohamed M Arafa, Ginevra Farnetani, Miguel J Xavier, Manon S Oud, Ahmad A Majzoub, Liliana Ramos, Chiara Abrardo, Matilde Spinelli, Daniel Moreno-Mendoza, Giuseppe Defazio, Elisabet Ars, Marc Pybus, Josvany R Sánchez Curbelo, Haitham T Elbardisi, Shoaib Nawaz, Najeeb Syed, Eduard Ruiz-Castané, Godfried W van der Heijden, Khalid A Fakhro, Joris A Veltman, Csilla Krausz

**Affiliations:** Department of Andrology, Fundació Puigvert, Universitat Autònoma de Barcelona, Instituto de Investigaciones Biomédicas Sant Pau (IIB-Sant Pau), Barcelona, Catalonia, Spain; Division of Genetics, Oregon National Primate Research Center, Oregon Health & Science University, Beaverton, OR, USA; Department of Urology, Hamad Medical Corporation, Doha, Qatar; Department of Clinical Urology, Weill Cornell Medicine-Qatar, Doha, Qatar; Department of Andrology, Cairo University, Cairo, Egypt; Department of Experimental and Clinical Biomedical Sciences “Mario Serio”, University of Florence, Florence, Italy; Faculty of Medical Sciences, Biosciences Institute, Newcastle University, Newcastle-upon-Tyne, UK; Department of Human Genetics, Donders Institute for Brain, Cognition and Behaviour, Radboudumc, Nijmegen, The Netherlands; Department of Urology, Hamad Medical Corporation, Doha, Qatar; Department of Clinical Urology, Weill Cornell Medicine-Qatar, Doha, Qatar; Department of Obstetrics and Gynaecology, Radboud University Medical Centre, Nijmegen, The Netherlands; Department of Andrology, Fundació Puigvert, Universitat Autònoma de Barcelona, Instituto de Investigaciones Biomédicas Sant Pau (IIB-Sant Pau), Barcelona, Catalonia, Spain; Department of Experimental and Clinical Biomedical Sciences “Mario Serio”, University of Florence, Florence, Italy; Department of Andrology, Fundació Puigvert, Universitat Autònoma de Barcelona, Instituto de Investigaciones Biomédicas Sant Pau (IIB-Sant Pau), Barcelona, Catalonia, Spain; Department of Urology, Hospital Francisco Grande Covián, Arriondas, Asturias, Spain; Department of Experimental and Clinical Biomedical Sciences “Mario Serio”, University of Florence, Florence, Italy; Department of Biosciences, Biotechnology and Environment, University of Bari Aldo Moro, Bari, Italy; Molecular Genetic Laboratory Fundació Puigvert, Universitat Autònoma de Barcelona, Instituto de Investigaciones Biomédicas Sant Pau (IIB-Sant Pau), Barcelona, Catalonia, Spain; Molecular Genetic Laboratory Fundació Puigvert, Universitat Autònoma de Barcelona, Instituto de Investigaciones Biomédicas Sant Pau (IIB-Sant Pau), Barcelona, Catalonia, Spain; Department of Andrology, Fundació Puigvert, Universitat Autònoma de Barcelona, Instituto de Investigaciones Biomédicas Sant Pau (IIB-Sant Pau), Barcelona, Catalonia, Spain; Department of Urology, Hamad Medical Corporation, Doha, Qatar; Department of Clinical Urology, Weill Cornell Medicine-Qatar, Doha, Qatar; Department of Clinical Urology, College of Medicine, Qatar University, Doha, Qatar; Laboratory of Reproductive Biology, Sidra Medicine, Doha, Qatar; Laboratory of Genomic Medicine, Sidra Medicine, Doha, Qatar; Laboratory of Genomic Medicine, Sidra Medicine, Doha, Qatar; Department of Andrology, Fundació Puigvert, Universitat Autònoma de Barcelona, Instituto de Investigaciones Biomédicas Sant Pau (IIB-Sant Pau), Barcelona, Catalonia, Spain; Department of Obstetrics and Gynaecology, Radboud University Medical Centre, Nijmegen, The Netherlands; Laboratory of Genomic Medicine, Sidra Medicine, Doha, Qatar; College of Health and Life Sciences, Hamad Bin Khalifa University (HBKU), Doha, Qatar; Department of Genetic Medicine, Weill Cornell Medicine, (WCM-Q), Doha, Qatar; Faculty of Medical Sciences, Biosciences Institute, Newcastle University, Newcastle-upon-Tyne, UK; Institute of Genetics and Cancer, College of Medicine and Veterinary Medicine, University of Edinburgh, Edinburgh, UK; Department of Experimental and Clinical Biomedical Sciences “Mario Serio”, University of Florence, Florence, Italy

**Keywords:** azoospermia, TESE, genetics, diagnostic, male infertility, spermatogenesis, exome, gene panel, POI

## Abstract

**STUDY QUESTION:**

What is the diagnostic yield and the pre-testicular sperm extraction (TESE) prognostic value of a non-obstructive azoospermia (NOA)-specific virtual gene panel?

**SUMMARY ANSWER:**

The diagnostic yield in our cohort was 6.1%, and by combining our data with published literature, we identified 11 genes compatible with testicular sperm production and 19 genes associated with no sperm retrieval in carriers of pathogenic (P) or likely pathogenic (LP) mutations.

**WHAT IS KNOWN ALREADY:**

Azoospermia, the most severe form of male infertility, affects ∼1% of the male population, with TESE being the primary treatment option. However, in NOA, TESE fails in nearly 50% of cases and existing clinical parameters are unable to predict TESE failure. Over the past decade, next-generation sequencing (NGS) has identified several candidate NOA genes, but their diagnostic utility and impact on TESE outcomes have not been fully explored.

**STUDY DESIGN, SIZE AND DURATION:**

A literature search was addressed to identify well-established NOA genes for designing a specific virtual gene panel for NOA. Our retrospective study analysed the diagnostic yield of the NGS-based virtual gene panel, comprising 145 genes, in 571 men affected by idiopathic NOA with known TESE outcomes. Subsequently, a second literature search was performed to identify carriers of LP/P variants in the genes where we identified mutations, focusing on individuals with known TESE outcomes. This approach allowed us to integrate the published data with our findings and predict a genotype–phenotype correlation between the affected genes and TESE success.

**PARTICIPANTS/MATERIALS, SETTINGS, METHODS:**

571 NOA patients with known TESE outcomes were recruited in two European and one Middle East centres. Variants were obtained from a whole-exome sequencing dataset and crossed with the 145 genes of the virtual gene panel. After a filtering process, variants were manually assessed and classified according to ACMG guidelines by using two methods: (i) In order to compare our data with previously published studies, we applied ACMG-AMP guidelines along with ClinGen recommendations used by other similar studies. (ii) A new approach was used to optimize ACMG-AMP guidelines with all ClinGen recommendations and incorporated NOA-specific rules addressing phenotypic, locus, and allelic heterogeneity. LP and P variants were confirmed by Sanger sequencing.

**MAIN RESULTS AND THE ROLE OF CHANCE:**

By using the new variant classification approach adapted for NOA, we identified LP/P variants in 6.1% of patients, with a higher yield (9.4%) in cases with negative TESE outcomes and maturation arrest (11.7%). By integrating our findings with the literature, we highlight 19 genes recurrently associated with negative TESE outcomes and 11 genes associated with positive sperm retrieval either in the testis or in semen. TESE is recommended for patients with LP or P variants in the 11 specific genes. Notably, six of these genes are located on the X chromosome, therefore, these variants will be obligatorily transmitted to daughters, and potentially increase the risk of NOA-related infertility in male offspring. We observed that nine genes, in which we identified LP/P variants, have been previously described in individuals with premature ovarian insufficiency (POI). Of these, eight were associated with negative TESE outcomes in men. Furthermore, we propose seven additional genes mutated in our cohort of NOA patients as novel POI candidates. These genes have not yet been considered as POI candidates, but they result in female infertility when knocked out in mouse models.

**LARGE SCALE DATA:**

LP/P variants have been submitted to ClinVar (https://www.ncbi.nlm.nih.gov/clinvar/).

**LIMITATIONS, REASONS FOR CAUTION:**

NOA is genetically heterogeneous, and our panel excludes those genes which were reported only in a single subject or single family. Although this can limit the diagnostic yield in our study, it ensures that only genes with clear relationship with NOA have been analysed. While in our cohort TESE outcomes are known for all patients, this information is often not available for mutation carriers in the published studies. Consequently, the total number of patients with P variants in the same gene remains relatively low, limiting our final conclusions. However, even if the number of carriers of genes associated with positive sperm retrieval is relatively low, it does not constrain our conclusions regarding TESE prediction. On the other hand, caution is warranted for genes linked to negative TESE outcomes, except for *TEX11, SYCE1*, and *MSH4*, each of which have 10 or more reported TESE-negative cases.

**WIDER IMPLICATIONS OF THE FINDINGS:**

Our study was performed on the largest available NOA cohort with known TESE outcomes. It not only provides an estimate on the diagnostic potential of a NOA-specific virtual gene panel, but it also advances the understanding of genetic factors influencing TESE outcomes. Half of the genes mutated in our study and presenting TESE-positive outcomes are already informative for clinical decision-making. The observed genotype–phenotype correlations may help in personalized decision-making prior to TESE, in order to undergo the procedure or to avoid unnecessary invasive treatment. It provides valuable insights that can inform clinical management strategies and potentially offer personalized treatments based on genetic profiles. The use of two different variant classification methods highlights that previous studies may have over-estimated the diagnostic yield, underscoring the need for a standardized variant classification approach addressed specifically to male infertility. Our study also emphasizes the overlap between NOA- and POI-associated genes, which has important clinical implications for genetic counselling of female siblings of affected individuals.

**STUDY FUNDING/COMPETING INTEREST(S):**

This work was funded by the Spanish Ministry of Health Instituto Carlos III-FIS FONDOS FEDER (grant numbers PI20/01562 and PI23/00425) and the Fanconi Research Fund awarded to C.K. and A.R.-E. This article is based upon work from COST Action CA20119 (ANDRONET), supported by COST (European Cooperation in Science and Technology) (www.cost.eu). C.K., A.R.-E., G.F., M.J.X., M.S.O., C.A., M.S., and E.R.-C. are members of the Action. This research was also supported by the Qatar National Research Fund (QNRF) under grant NPRP12S-0318-190394, and by an Investigator Award in Science from the Wellcome Trust (209451 to J.A.V.). The authors declare no competing interests.

WHAT DOES THIS MEAN FOR PATIENTS? Non-obstructive azoospermia (NOA) is the most severe form of male infertility where spermatozoa are not found in the ejaculate. It affects about 1% of men in the general population and, in more than 90% of cases, it is caused by a problem in the testicles themselves. The only chance of having a biological child is through surgery to try to retrieve sperm directly from the testicles, a procedure called testicular sperm extraction (TESE), followed by IVF. However, in nearly half of the cases, TESE does not succeed in finding any sperm, and doctors still cannot reliably predict in advance who will benefit from this procedure. Over the past 10 years, a new DNA testing method called next-generation sequencing (NGS) has helped to identify several genes linked to NOA. However, we do not yet fully know how useful this genetic information is for diagnosing the condition or predicting whether TESE surgery will be successful.

We looked at the genetic information of 571 men with azoospermia from different ethnic backgrounds, all of whom had undergone TESE, and we tested 145 genes known to be linked to sperm production. We found that patients who had no sperm recovery or those with testicular tissue showing signs of no mature sperm production had significantly higher chances of being carriers of a genetic alteration. Additionally, by integrating our findings with existing literature, we identified specific genes in which likely pathogenic and pathogenic variants were linked to successful sperm production, as well as those associated with failed sperm production. In this study, we also provide a new method specifically developed for the interpretation of genetic findings in the context of NOA.

## Introduction

Male infertility is a multifactorial and heterogeneous pathological condition affecting 7% of the male population. The most severe form is non-obstructive azoospermia (NOA), which occurs in ∼1% of men in the general population (Krausz and Riera-Escamilla, 2018). Current genetic diagnostic tests for NOA include karyotyping and Y chromosome azoospermia factor (AZF) microdeletion analysis. These analyses provide a genetic diagnosis in about 15–20% of men with NOA and have important clinical implications for patient management to such an extent that they can be used for decision-making in performing a testicular sperm extraction (TESE) procedure. For example, patients with complete AZFa or AZFb/bc deletions should be counselled not to undergo TESE since the likelihood of retrieving spermatozoa is virtually zero (Krausz *et al.*, 2024). Conversely, a recent systematic review and meta-analysis has indicated that the sperm retrieval success rates in patients with AZFc deletions is around 40%, similar to the success rate in those with Klinefelter syndrome (Majzoub *et al.*, 2022). For those patients for whom the cause of their azoospermia remains unexplained (referred to as ‘idiopathic’), TESE remains the only option offered to them to potentially father a biological child. In these cases, TESE typically has a sperm recovery success rate of around 50% (Corona *et al.*, 2019).

The genetic landscape of male infertility is highly complex, with at least 2000 genes predicted to be involved in spermatogenesis (Hochstenbach and Hackstein, 2000). Over the past decade, thanks to the application of whole-exome sequencing (WES), hundreds of monogenic causes of male infertility have been reported in NOA. Due to the extensive genetic heterogeneity of azoospermia, the reported genes are often mutated in only a single individual; therefore, only a limited number of genes have sufficient clinical evidence to justify their inclusion in a diagnostic gene panel (Oud *et al.*, 2019; Houston *et al.*, 2021; Riera-Escamilla and Nagirnaja, 2025). Furthermore, the interpretation of a variant’s pathogenicity is not always straightforward, as various approaches with varying levels of stringency have been described. Typically, criteria with lower stringency may result in a higher number of false-positive (likely) pathogenic variants. This is particularly significant as variant interpretation influences clinical decision-making.

To close this gap, we have analysed a virtual gene panel encompassing the 145 genes with at least limited gene–disease relationship (GDR) evidence identified in a literature search, and for which the prognostic value in predicting TESE outcomes is still unknown. We analysed this gene panel in a cohort of 571 men affected by idiopathic NOA and with known TESE outcomes. This study had two distinct purposes. First, we wanted to evaluate the capacity of this gene panel in identifying monogenic causes of NOA and determine its diagnostic yield. Second, we sought to determine which genes, when mutated, are compatible with testicular sperm production and which are not. This latter aim was intended to support clinicians and patients in making informed decisions regarding the advisability of an invasive procedure such as TESE.

## Materials and methods

### Subjects

#### Infertile men

A total of 571 patients with NOA and known outcome of TESE (positive or negative sperm recovery) were enrolled in this study. Individuals with known acquired, e.g. chemotherapy, orchitis, testicular torsion, etc. and genetic (karyotype aberrations, Y chromosomal AZF deletions) causes of azoospermia were excluded. Among the 571 patients, 262 were enrolled in Barcelona (Spain), 109 were enrolled in Doha (Qatar), and 200 were enrolled in Newcastle (UK) and Nijmegen (The Netherlands) (Information on TESE procedures is provided in the Supplementary Materials and Methods and Supplementary Fig. S1 shows a schematic representation of our study design). All subjects, and family members where applicable, provided written informed consent to be included in the study. The study protocol was approved by the local ethics committees (Barcelona: Fundació Puigvert: 2014/04c; Doha: MRC-03-21-065; Nijmegen: NL50495.091.14 version 5.0; Newcastle: Ref: 18/NE/0089, Bursa: 05.01.2015/04).

#### Normozoospermic control individuals

Exome data from 311 normozoospermic control individuals enrolled in Barcelona, prior to a vasectomy procedure, were used for improving variant classification. All controls were normozoospermic based on total sperm count, total motile sperm count, and total number of spermatozoa with typical morphology. All of them achieved natural pregnancy within 12 months of attempting conception. The distribution of their main sperm parameters is provided in Supplementary Table S1. All normozoospermic controls provided written informed consent. The study protocol was approved by the local ethics committees (Barcelona: Fundació Puigvert: 2014/04c).

### Virtual gene panel design

We prepared a list of 145 genes involved in NOA or severe oligozoospermia reported in PubMed until 1 January 2023. These genes were selected according to the following inclusion criteria: (i) genes with at least limited evidence for azoospermia reported in Houston *et al.* (2021) (N = 48); and (ii) genes mutated in more than one unrelated patient with NOA, not included in the Houston *et al.* review (N = 97). Among the 145 genes, 89 are associated with autosomal recessive inheritance, 41 are X-linked genes, 2 are Y-linked, and 13 are reported to be associated with dominant inheritance in the context of male infertility (Supplementary Table S2 and Supplementary Fig. S1).

### Exome and genome sequencing and variant annotation

WES samples were prepared and enriched as previously described in Krausz *et al.* (2020) for the Barcelona cohort and Oud *et al.* (2022) for the Nijmegen/Newcastle cohort. There were 43 patients from the Nijmegen/Newcastle cohort who underwent whole-genome sequencing (WGS) (Supplementary Materials and Methods), and 69 patients from Doha underwent WES and samples were prepared as described in Fakhro *et al.* (2018), whereas the remaining 90 samples from Doha underwent WGS as described in Abdi *et al.* (2023) (see Supplementary Materials and Methods for further details). All variants were annotated using the canonical transcript in Ensembl Variant Effect Predictor release 112 (VEP; McLaren *et al.* 2016).

Copy number variations (CNVs) were identified using the following tools: ExomeDepth (Plagnol *et al.*, 2012) for samples from Barcelona, CNVRobot (https://github.com/AnetaMikulasova/CNVRobot) and dysgu-sv (Cleal and Baird, 2022) for samples from Newcastle/Nijmegen, and using a combinatorial approach as described in Rossi *et al.* (2021) for samples from Doha (see Supplementary Materials and Methods for further details).

### Variant filtration

We selected non-synonymous exonic and splicing variants (within ±3 positions of the splice site) and with a minor allele frequency (MAF) ≤0.01 (for autosomal-recessive inheritance) and ≤0.001 (for autosomal-dominant, X- and Y-linked inheritance) based on the maximum population allele frequency observed in the genome aggregation database (gnomAD v4.1.0; https://gnomad.broadinstitute.org/). We filtered out variants with <10× depth of coverage and with a ratio of mutated/all reads <0.15. We took into consideration only those variants which are in accordance with the expected mode of inheritance (recessive, dominant, and sex-linked). Concerning CNVs, we selected homozygous or heterozygous deletions or predicted to be in trans with another SNV or CNV (compound heterozygous) in a gene associated with autosomal recessive inheritance. We filtered out common deletions with similar breakpoints identified in >10 unrelated carriers or with MAF ≥ 1% for genes associated with autosomal recessive inheritance and MAF ≥ 0.1% for sex-linked genes, as reported in gnomAD SVs v4.1.0 (https://gnomad.broadinstitute.org/).

### Variant classification

In this study, we classified variants by applying two different methods. First, to compare our data with previously published studies, we classified variants adapting the criteria described by Wyrwoll *et al.* (2023). In their study, Wyrwoll *et al.* (2023) applied the ACMG-AMP guidelines (Richards *et al.*, 2015) along with certain ClinGen recommendations (https://www.clinicalgenome.org/). Second, we classified variants according to the most recent standards in diagnostic variant classification including ACMG-AMP guidelines, optimized using ClinGen recommendations, as well as NOA-specific rules incorporating phenotypic, locus, and allelic heterogeneity (Supplementary Materials and Methods). Consequently, this method is more stringent and therefore minimizes potential overestimation of likely pathogenic (LP) and pathogenic (P) variants leading to a different diagnostic yield.

For both classifications, we used the scaled point system described by Tavtigian *et al.* (2020) to classify each variant according to the total score obtained: (i)  ≥ 10: P; (ii)  ≥ 6 and ≤9: LP; (iii)  ≥ 0 and ≤5: variant of uncertain significance (VUS); (iv)  ≥ −6 and ≤−1, likely benign (LB); and (v) ≤−7, benign (B).

### Validation of the selected variants

We validated all variants classified as LP or P through Sanger sequencing, qPCR, and +/− PCR, except for seven variants for which DNA was unavailable. For these variants, we examined the Integrative Genomics Viewer (Robinson *et al.*, 2011) (Supplementary File S1).

### Evaluation of the relationship between genes and TESE outcomes

To evaluate whether a given gene was systematically mutated in specific TESE outcomes, we conducted a literature search for studies reporting variants in genes in which we identified LP/P variants based on the variant classification criteria adapted from Wyrwoll *et al.* (2023). This search included publications up to and including 1 June 2024 based on the PubMed electronic database. Patients with spermatozoa in the ejaculate (extreme oligozoospermia—from >0 to <2 million sperm—or severe oligozoospermia from >2 to <10 million) were classified as TESE-positive. To evaluate potential differences in the biological roles of genes exclusively associated with TESE-negative outcomes versus those associated with both TESE-positive and TESE-negative outcomes, we used the Cytoscape plug-in ClueGO (Shannon *et al.*, 2003), the human infertility single-cell testis atlas (HISTA) (Mahyari *et al.*, 2025), and Mouse Genome Informatics database (https://www.informatics.jax.org/) and references therein.

### Assessment of GDR

We assessed the GDR for the 40 genes with variants classified as LP/P by Wyrwoll *et al.* (2023) criteria according to the method used by Houston *et al.* (2021).

### Statistical analyses

Statistical analyses were performed using R software (R Core Team, 2024). To test whether the frequency of LP/P, VUS, or LB/B variants was more prevalent in patients with negative TESE outcomes, the number of patients with specific variants was recorded. A contingency table was created to compare the presence of variants between the TESE+ and TESE− groups. Odds ratios (OR) and their 95% CIs were calculated using the Wald method. Binomial exact test was applied with the null hypothesis that given the LP/P, VUS, or LB/B variants frequency, P(TESE+) is equal to P(TESE−) and a two-sided alternative hypothesis was assessed. Regarding differences in the detection of LP/P based on testicular phenotype, chi-square tests were used to assess the differences in the frequency of P variants between pairs of groups (Sertoli cell only (SCO) vs maturation arrest (MA), SCO vs hypospermatogenesis (HSG), and MA vs HSG). To correct for multiple comparisons, *P*-values were adjusted using the Bonferroni method, and the statistical significance of these comparisons was evaluated using only the adjusted *P*-values.

## Results

### Clinical characteristics of the study cohort

A group of 571 idiopathic men affected by NOA from three different cohorts participated in this study. All patients underwent TESE, and successful sperm recovery was achieved in 242 patients (42.4%), whereas for the remaining 329 patients (57.6%), the outcome of TESE was negative. Based on the most advanced cell type observed in the testis histology, 238 patients were affected by SCO, 171 by MA (at the spermatogonia, spermatocyte, or spermatid level), and 143 were affected by HSG, and six by Tubular Shadows (TS), in which complete hyalinization was observed. Testis histology descriptions were not available for 13 men. The clinical characteristics of the patients are reported in Supplementary Table S3.

### Identification of potential genetic causes of NOA

#### Detection rate of candidate variants based on previously published variant classification approach

**Figure 1.**
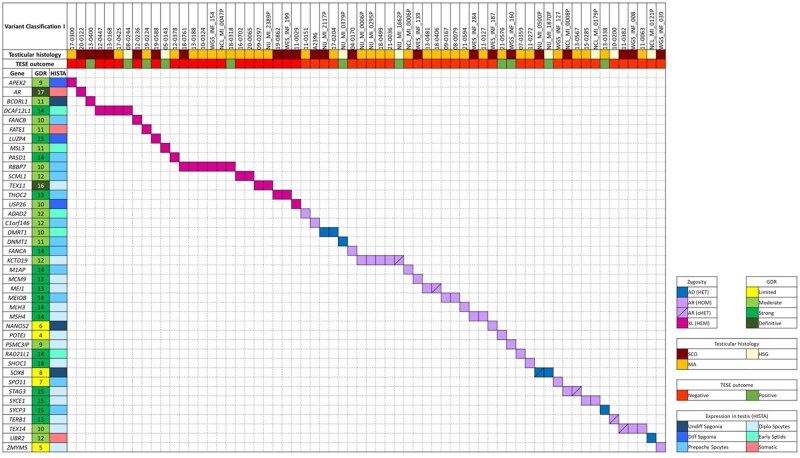
**Description of the (likely) pathogenic variants reported in the current study identified by using the ACMG/AMP-based variant interpretation framework, adapted from**  **Wyrwoll *et al.* (2023) (Classification I).** For each carrier, the testicular histology (if available) and TESE outcome are reported; the GDR and expression of each gene in the testis are also reported. Abbreviations: TESE, Testicular Sperm Extraction; GDR, Gene–Disease Relationship; HISTA, Human Infertility Single-cell Testis Atlas; AD, autosomal dominant; AR, autosomal recessive; XL, X-linked; HET, heterozygous; HOM, homozygous; cHET, compound heterozygous; HEM, hemizygous; SCO, Sertoli-cell only; MA, maturation arrest; HSG, hypospermatogenesis; Undiff Spgonia, undifferentiated spermatogonia; Diff Spgonia, differentiated spermatogonia; Prepachy Spcytes, pre-pachytene spermatocytes; Diplo Spcytes, diplotene spermatocytes; Early Sptids, early spermatids.

To identify LP and P genetic variants that could explain the infertile phenotype of our patients and to compare our findings with those reported in the literature, we first classified all genetic variation detected by our virtual gene panel according to the ACMG-AMP guidelines by adapting the criteria described by Wyrwoll *et al.* (2023). By applying this criteria, we identified a total of 38 variants classified as P, 33 as LP, 246 as VUS, 6 as LB, and 3 as B. In total, we identified 64 patients carrying 70 LP/P variants in infertility genes included in the virtual panel, reflecting a detection rate of LP/P variants of 11.2% (Supplementary Table S4 and Fig. 1). Of these LP/P variants, 27 were homozygous, 25 were hemizygous, 10 were compound heterozygous (validated by sequencing the DNA from their parents and relatives, data not shown), and 8 were heterozygous in genes associated with dominant inheritance. In our study, we have not observed differences in the detection rate of LP/P variants between patients from regions where consanguineous marriages are relatively frequent and those from Europe or North America where consanguinity is rare (13.55% vs 9.77%). In patients from geographic regions where consanguinity is prevalent (such as Middle East, Morocco, India, Pakistan etc.), the majority of LP/P variants were homozygous in genes associated with autosomal recessive inheritance (19/28 cases with LP/P variants). In contrast, patients from countries where consanguinity is less prominent showed a lower prevalence of homozygous LP/P variants in recessive disease genes (7/35 cases with LP/P variants). Interestingly, half of the patients from regions with low consanguinity (17/35 cases) had the LP/P variant mapped to the X chromosome, despite the fact that only one-third of the genes included in the gene panel are X-linked.

The 70 LP/P variants affected 40 genes (Supplementary Table S4 and Fig. 1). Among these, 14 genes (*DCAF12L1, DMRT1, KCTD19, MEI1, MEIOB, MSH4, RBBP7, SCML1, SOX8, STAG3, SYCE1, TEX11, TEX14, THOC2)* were found to be mutated in more than one azoospermic man. Interestingly, in 9 of these recurrently affected genes (*DMRT1, KCTD19, MSH4, RBBP7, STAG3, SYCE1, TEX11, TEX14, THOC2)*, the affected carriers belonged to different centres. The most frequently affected gene is the X-linked gene *RBBP7*, which was found to be mutated in six patients across the three cohorts. The second most affected gene is *KCTD19*, in which a total of five patients carried LP/P variants in our study cohort. *DCAF12L1* is the third most affected gene, found to be mutated in four patients. Interestingly, in three of these patients, the P mutation is a complete gene deletion. Three infertile men carried LP/P mutations in *TEX14*. The remaining 10 recurrently affected genes (*DMRT1, MEI1, MEIOB, MSH4, SCML1, SOX8, STAG3, SYCE1, TEX11, THOC2)* were found to be mutated in two unrelated cases.

Concerning the 246 variants classified as VUS, they were located in 75 genes and not considered causative. However, within this group, 53 variants are predicted to be P by most of the prediction tools. Consequently, future functional assays and the identification of additional patients carrying the same variants will be essential to reclassify them as LP, consequently increasing the overall diagnostic yield of a gene panel approach.

#### Diagnostic yield according to ACMG/AMP-based variant interpretation framework refined to genetic diagnostics in NOA

While acknowledging the previously published variant classification criteria by Wyrwoll *et al.* (2023), which have made adaptions of the ACMG/AMP criteria in accordance with recent recommendations (including those of the ClinGen Sequence Variant Interpretation Working Group), several evidence criteria were nonetheless applied more liberally than is generally accepted in clinical genetic laboratories.

Additionally, these adaptations were not specifically tailored to NOA. This can result in overestimation of the P effect of a variant. To provide a more conservative pathogenicity score and diagnostic yield in line with the practice of clinical genetic diagnostic laboratories, we set up a framework for sequence variant classification specified to NOA.

**Figure 2.**
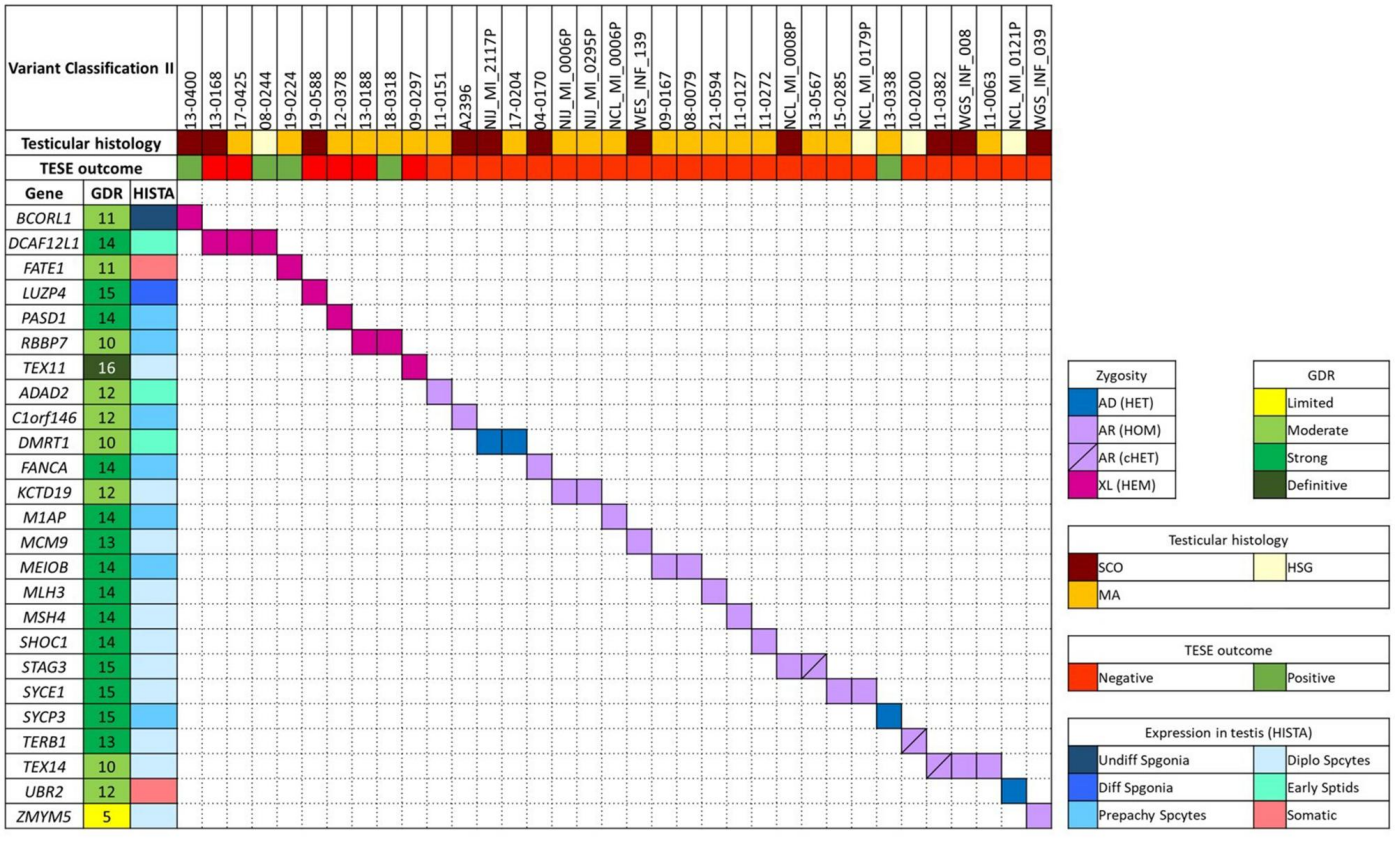
**Description of the (likely) pathogenic variants reported in the current study identified by using the ACMG/AMP-based variant interpretation framework refined to genetic diagnostics in non-obstructive azoospermia (Classification II).** For each carrier, the testicular histology (if available) and TESE outcome are reported; the GDR and expression of each gene in the testis are also reported. Abbreviations: TESE, Testicular Sperm Extraction; GDR, Gene–Disease Relationship; HISTA, Human Infertility Single-cell Testis Atlas; AD, autosomal dominant; AR, autosomal recessive; XL, X-linked; HET, heterozygous; HOM, homozygous; cHET, compound heterozygous; HEM, hemizygous; SCO, Sertoli-cell only; MA, maturation arrest; HSG, hypospermatogenesis; Undiff Spgonia, undifferentiated spermatogonia; Diff Spgonia, differentiated spermatogonia; Prepachy Spcytes, pre-pachytene spermatocytes; Diplo Spcytes, diplotene spermatocytes; Early Sptids, early spermatids.

A total of 24 variants were classified as P, 15 as LP, 139 as VUS, 113 as LB, and 35 as B (Supplementary Table S4). In total, we identified 35 patients carrying LP/P variants, resulting in a diagnostic yield of 6.1% (Fig. 2). Among these patients, the causative variants were distributed as follows: 18 were homozygous, 10 were hemizygous, 6 were compound heterozygous (validated by sequencing the DNA from their parents and relatives, data not shown), and 4 were heterozygous within genes associated with dominant inheritance. Similar to the findings obtained using the first classification criteria, the diagnostic yield was not higher in patients from countries with a higher prevalence of consanguinity. However, there was a notable overrepresentation of homozygous variants among patients from these geographic regions, with 13 of 19 patients harbouring homozygous variants.

The LP/P variants impacted 25 genes: *ADAD2, BCORL1, C1orf146, DCAF12L1, DMRT1, FANCA, FATE1, KCTD19, LUZP4, M1AP, MCM9, MEIOB, MLH3, MSH4, PASD1, RBBP7, SHOC1, STAG3, SYCE1, SYCP3, TERB1, TEX11, TEX14, UBR2*, and *ZMYM5.* Eight genes were recurrently mutated in more than one patient: *DCAF12L1*, *DMRT1*, *KCTD19*, *MEIOB*, *RBBP7*, *STAG3*, *SYCE1*, and *TEX14*. The most frequently affected genes were *DCAF12L1* and *TEX14*, each with three carriers. Interestingly, for *TEX14*, carriers were recruited from different centres, while all three patients with mutations in *DCAF12L1* were recruited in Barcelona, but their origins spanned different countries (Spain and Germany). Regarding the 139 variants classified as VUS, 34 are of potential interest for follow-up with further functional analysis, as they belong to four categories: splice variants (potentially affecting splice acceptors/donors, despite having SpliceAI scores lower than 0.9), stop gains, frameshifts (removing less than 10% of the protein without affecting any known domain), and missense variants with a REVEL score greater than 0.644.

### Similarities and differences between the output of the two classification methods

The two methods result in different numbers of genes with LP or P variants, with the refined genetic diagnostic approach for NOA identifying fewer such genes. Specifically, 32 variants previously classified as LP/P were reclassified as VUS. Of these, most (25/32) were missense variants, while 8 were predicted as loss-of-function variants (LoF, e.g. splicing, start loss, stop gain, and frameshift variants). Additionally, 19 of the 32 reclassified VUS variants were located in X-linked or genes associated with dominant inheritance. In contrast, 40 variants remained classified as LP/P, the majority of which (n = 38) were predicted to be LoF. While the Wyrwoll *et al.* (2023) method is highly valuable in the research context of androgenetics, these results highlight the importance of applying the adapted diagnostic method for patient management.

### Upgrading the GDR

We have calculated the GDR according to the method used in Houston *et al.* (2021), therefore, for this part of the study, we used data on the 40 genes with LP/P variants. Among them, 35 reached the level of ‘moderate’ or above (Fig. 1). In 13 out of 35 genes, the GDR were considered below ‘moderate’ in Houston *et al.* (2021) and as a result of our re-assessment, based on novel carriers in our cohort and in the literature, they have been upgraded to ‘moderate’ or above. In addition, 4/35 genes (*FANCA*, *PASD1*, *STAG3*, *SYCE1*) previously reported as ‘moderate’ in the Houston *et al.* review (Houston *et al.*, 2021) have reached the level of ‘strong’ clinical evidence.

### Patients with LP/P variants have a significant likelihood of negative TESE outcomes

In our overall cohort, the sperm retrieval rate was 42%. We observed a significantly higher number of patients carrying LP/P variants in the 145 tested genes among those with negative TESE outcomes, regardless of the variant classification method employed, compared to those in whom sperm recovery was successful. Among the 64 patients carrying LP/P variants based on the first type of classification, only 11 had successful sperm recovery. Similarly, only 5 men out of 35 men with LP/P variants classified with the second diagnostic method had sperm recovered by TESE. Therefore, we found a significant association between being a carrier of an LP/P variant and a negative TESE outcome (Table 1). The higher frequency of LP/P variants in patients with negative TESE outcomes was also observed when analysing the three cohorts separately (Barcelona, Newcastle/Nijmegen and Doha). Such associations were not observed when we compared the frequencies of B, LB, or VUS with respect to TESE outcome, suggesting that only LP/P variants may play a significant role in determining negative TESE outcomes (Supplementary Table S5).

**Table 1.** Summary of the comparisons performed on carriers of LP/P variants according to two different ACMG-AMP variant classification methods; comparisons were performed in function of TESE outcome or testis phenotype.

		Carriers of LP/P variants according to different classification methods n (%)	
	**Entire cohort** (n = 571)	**Variant classification I** (n = 64)	**Variant classification II** (n = 35)
*TESE outcome*	Negative (n = 329, 57.6%)	53 (16.11)	30 (9.11)
	Positive (n = 242, 42.4%)	11 (4.51)	5 (2.06)
	Negative vs positive *P*-value, OR (CI 95%)	6.03 × 10^−8^***, 4.13 (2.11–8.08)	1.29 × 10^−5^***, 4.99 (1.89–12.9)
*Testis phenotype*	MA (n = 171)	34 (19.9)	20 (11.7)
	SCO (n = 238)	22 (9.2)	11 (4.6)
	HSG (n = 143)	8 (5.6)	4 (2.8)
	MA vs SCO *P*-value, OR (IC 95%)	0.0158*, 2.31 (1.26–4.29)	0.0680, 2.48 (1.12–5.75)
	MA vs HSG *P*-value, OR (IC 95%)	0.0011**, 4.2 (1.83–10.89)	0.0173*, 4.62 (1.5–19.03)

Odds ratios (OR) with 95% CIs were calculated using the Wald method. The binomial exact test was used to compare variant frequencies between TESE-positive and TESE-negative groups under a two-sided hypothesis. Differences in the frequency of LP/P variants among testicular phenotypes (SCO, MA, HSG) were assessed using chi-square tests. *P*-values from pairwise comparisons (SCO vs MA, SCO vs HSG, MA vs HSG) were adjusted for multiple testing by the Bonferroni method. Statistical significance is indicated by asterisks as follows: *P* < 0.05;

** *P* < 0.01;

*** *P* < 0.001. Statistically significant results are highlighted in **bold**.

ACMG, American College of Medical Genetics; AMP, Association for Molecular Pathology; Variant classification I: ACMG/AMP-based, adapted from Wyrwoll *et al.* (2023); Variant classification II: ACMG/AMP-based variant interpretation framework refined to genetic diagnostics in non-obstructive azoospermia; LP/P, likely pathogenic/pathogenic; VUS, variant of uncertain significance; LB/B, likely benign/benign; TESE, testicular sperm extraction; OR, odds ratio; SCO, Sertoli cell-only; MA, maturation arrest; HSG, hypospermatogenesis.

**Figure 3.**
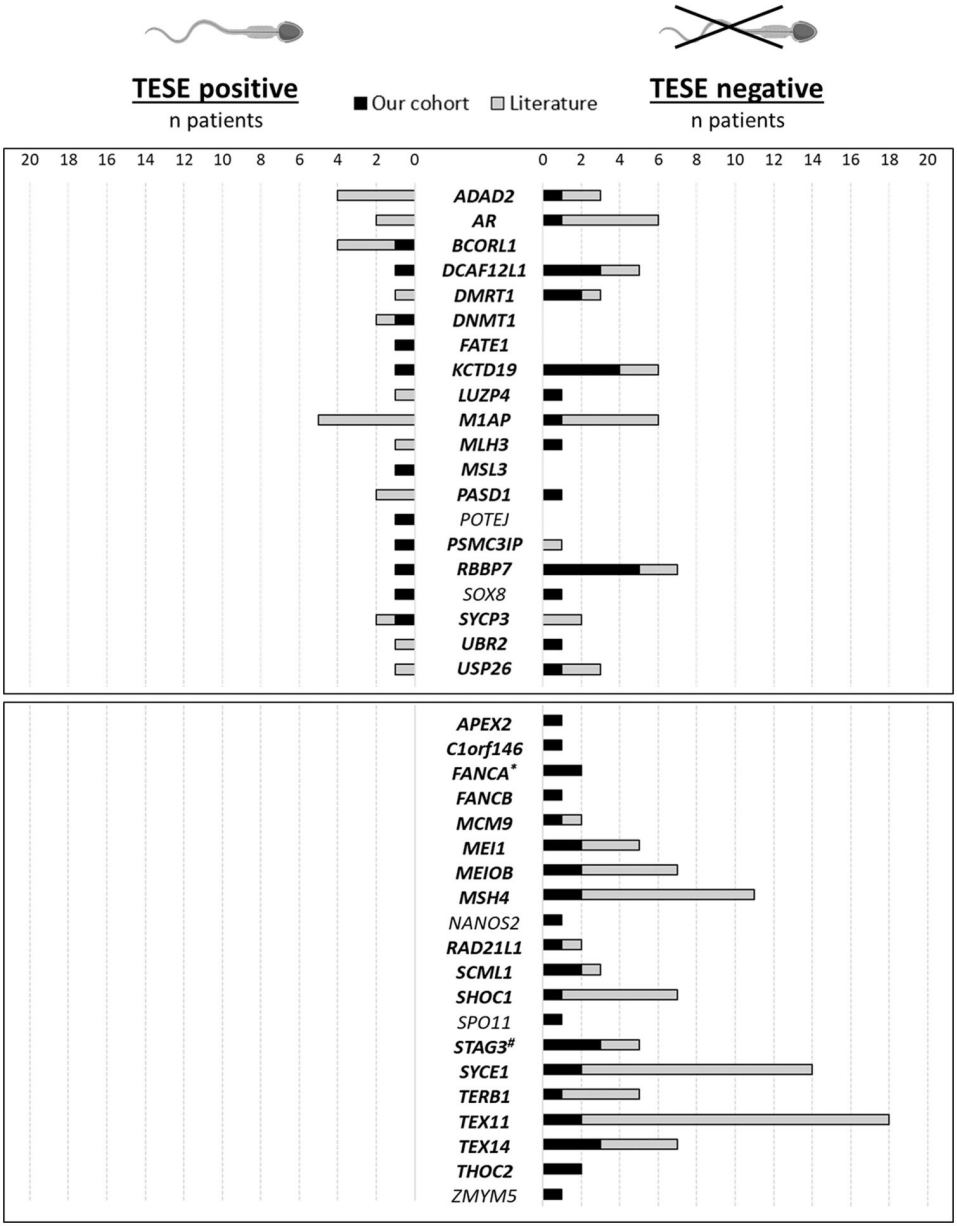
**Distribution of carriers of (likely) pathogenic variants, classified according to the ACMG/AMP-based variant interpretation framework, adapted from**  **Wyrwoll *et al.* (2023) (Classification I).** Distribution of carriers of (likely) pathogenic (LP/P) variants across our cohort (black bars) and the literature (grey bars), categorized by successful sperm retrieval (TESE-positive outcome, on the left) or unsuccessful sperm retrieval (TESE-negative outcome, on the right). The bars represent the number of carriers of LP/P variants for each gene, classified according to the ACMG/AMP-based variant interpretation framework, adapted from Wyrwoll *et al.* (2023) (Classification I). Genes in bold reach at least moderate Gene–Disease Relationship. An asterisk (*) indicates a patient identified as a FANCA mutation carrier, while a pound sign (#) indicates a patient identified as a STAG3 mutation carrier. Both patients were identified using a targeted gene panel in previous publications (Krausz *et al*., 2019; Riera-Escamilla *et al.*, 2019).

**Figure 4.**
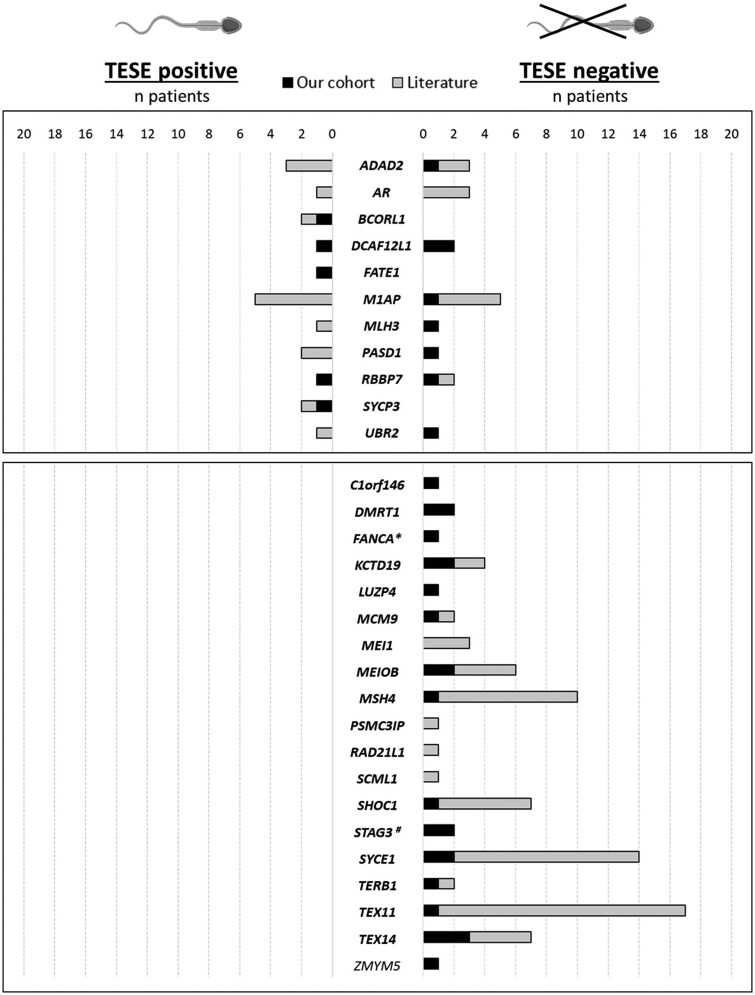
**Distribution of carriers of (likely) pathogenic variants, classified according to the ACMG/AMP-based variant interpretation framework refined to genetic diagnostics in non-obstructive azoospermia (Classification II).** Distribution of carriers of (likely) pathogenic (LP/P) variants across our cohort (black bars) and the literature (grey bars), categorized by successful sperm retrieval (TESE-positive outcome, on the left) or unsuccessful sperm retrieval (TESE-negative outcome, on the right). The bars represent the number of carriers of LP/P variants for each gene, classified according to the ACMG/AMP-based variant interpretation framework refined to genetic diagnostics in non-obstructive azoospermia (Classification II). Genes in bold reach at least moderate Gene–Disease Relationship. An asterisk (*) indicates a patient identified as a FANCA mutation carrier, while a pound sign (#) indicates a patient identified as a STAG3 mutation carrier. Both patients were identified using a targeted gene panel in previous publications (Krausz *et al*., 2019; Riera-Escamilla *et al.,* 2019).

To determine whether the association between LP/P variants and TESE outcomes was supported by existing literature, we conducted a review of studies reporting on infertile men with rare variants in the 40 genes where we identified LP/P variants using the first classification criteria. After excluding patients with unknown TESE outcomes and variants re-classified as VUS or LB, we compiled a list of 111 patients carrying 124 LP/P variants. These variants were then further re-assessed using the diagnostic method, resulting in a list of 82 patients carrying 91 LP/P variants (Supplementary Tables S6 and S7). Consistent with the findings in our cohort, sperm recovery after TESE was unsuccessful in the majority of cases with LP/P variants (79.3% and 82% based on the less and more stringent variant classification methods, respectively; Figs 3 and 4). This finding further corroborates that a monogenic cause is more likely to be associated with a decreased TESE retrieval rate.

### Increased prevalence of LP/P variants in patients with MA

Next, we wanted to explore whether the frequency of monogenic factors varies based on testis histology. We observed a significantly higher diagnostic yield in patients with MA compared to those affected by SCO syndrome or HSG, regardless of the variant classification method employed. Specifically, using the first classification method, we found that 34/171 (19.9%) of patients with MA carried LP/P variants, whereas this percentage decreased to 9.2% (22/238) in patients with SCO and to 5.6% (8/143) in patients with HSG in our cohort. When applying the diagnostic variant classification method, we still observed differences in diagnostic yield between MA versus SCO (11.7% vs 4.6%, 20 and 11 cases, respectively) and MA versus HSG (11.7% vs 2.8%—20 vs 4 cases, respectively) (see Table 1).

### Predictive value of affected genes for TESE outcome: combined analysis with literature data

To determine which of the 40 genes in which we identified LP/P variants by using the less stringent criteria were consistently affected in negative TESE outcomes and which were compatible with sperm production, we combined genotype and phenotype data from our patients with those reported in the literature. By classifying variants, we observed that mutations in 20/40 genes were systematically associated with negative TESE outcomes, meaning no sperm were recovered in any patient. In contrast, for the remaining 20 genes, sperm were successfully recovered in some carriers (Fig. 3 and Supplementary Table S8).

When we applied the diagnostic criteria, we identified 19 genes where LP/P variants were reported only in patients with negative TESE outcomes and 10 genes where sperm were recovered in some instances. Notably, four genes (*DMRT1, KCTD19, LUZP4, and PSMC3IP*) that were previously linked to positive TESE in four cases were now restricted to negative TESE outcomes since the variants in the carriers did not fulfil the LP/P criteria. Additionally, applying the more stringent criteria resulted in the removal of five genes (*APEX2, FANCB, NANOS2, SPO11*, and *THOC2*) previously linked to negative TESE outcomes, and five genes (*USP26, SOX8, DNMT1, MSL3, and POTEJ*) previously associated with sperm recovery, as all their reported LP/P variants became VUS (Fig. 4 and Supplementary Table S9).

It is important to note that although the majority of these genes have at least moderate GDR evidence and therefore should be sequenced as part of the diagnostic routine, for most of them, the total number of patients carrying LP/P variants with known TESE outcomes remains small. For example, in 26 out of the 30 genes with LP/P variants classified with the diagnostic method, the number of patients with variants and known TESE outcomes is fewer than 10, While having a low number of carriers does not change the prediction, if at least one of the carriers have a positive TESE outcome, it does challenge the ability to predict negative TESE outcomes based on the affected gene. However, in 4 out of the 30 genes, the number of carriers with known TESE outcomes is  ≥ 10 (*M1AP, MSH4, SYCE1*, and *TEX11*). This relatively larger sample size helps to reveal the likelihood of sperm recovery depending on the affected gene. For instance, sperm were not recovered in any patient carrying LP/P variants in *TEX11* (n = 17), *SYCE1* (n = 14), and *MSH4* (n = 10), indicating that the likelihood of obtaining sperm in patients with LP/P variants in these genes is close to zero. On the other hand, the proportion of cases with successful sperm recovery is up to 50% for patients carrying *M1AP* LP/P mutations, as classified using the diagnostic method, which is the same likelihood observed in idiopathic NOA cases (Supplementary Table S9).

### Biological differences between genes linked to negative and positive TESE outcomes

To assess whether the genes associated with negative TESE outcomes differ biologically from the genes whose mutations are compatible with testicular sperm production, we analysed the biological processes they are involved in, their expression patterns in the testis, and the reproductive phenotypes of knockout (KO) mice (when available). Overall, as expected, we observed that both variant classifications methods yielded similar results. We used the Cytoscape plug-in ClueGO to compare the Gene Ontology biological processes enriched in each list of genes. Both groups of genes show enrichment in biological processes related to meiosis (Supplementary Figs S2 and S3).

When comparing the mRNA expression patterns described in HISTA, we observed that genes strictly associated with negative TESE outcomes are predominantly expressed in early germ cells (spermatogonia and spermatocytes). In contrast, the expression patterns of genes associated with successful sperm retrieval are more heterogeneous. For instance, the expression of *AR*, *UBR2*, and *FATE1*, all compatible with sperm production, is restricted to somatic testicular cells (Leydig and Sertoli cells). Similarly, *ADAD2* and *DCAF12L1*, also associated with TESE-positive outcomes, show mRNA expression in spermatids, suggesting a potential role in later spermatogenic stages. Moreover, when comparing the two gene lists, we found that only genes associated with negative TESE outcomes show a significant enrichment of genes differentially expressed in pre-pachytene germ cells (Component 51) (Mahyari *et al.*, 2025).

Regarding the data on KO mouse reproductive phenotypes, this also revealed slight differences between the two groups of genes. In >80% of the genes associated with negative TESE outcomes, KO mice are unable to produce mature spermatids, recapitulating the TESE-negative phenotype observed in men. Conversely, in >60% of genes associated with positive TESE outcomes in humans, KO mice can produce (few) spermatids or spermatozoa (Supplementary Tables S8 and S9).

## Discussion

Although NOA uniformly presents as the absence of spermatozoa in semen analysis, at the testicular level, its pathohistology is variable. This ranges from the complete lack of germ cells (tubular shadows and SCO) to the less severe phenotype characterized by the presence of few tubules with spermatozoa (HSG). It implies a complex genetic architecture underlying NOA, with a multitude of different genetic defects capable of disrupting different phases of spermatogenesis (Houston *et al.*, 2021).

NOA patients may achieve fertilization through ICSI using spermatozoa retrieved from the testis. TESE is an invasive procedure which is successful only in about 50% of cases (Corona *et al.*, 2019), prompting efforts to identify predictive factors. So far, no hormonal or clinical parameters can reliably predict TESE success. Only the diagnosis of complete AZFa or AZFb deletions (after defining the extension according to the latest EAA/EMQN guidelines) is associated with virtually zero chance of finding testicular spermatozoa (Krausz *et al.*, 2024). However, the frequency of AZF deletions in NOA ranges from 3% to 10% in different studies, implying that for a large majority of patients, we cannot offer personalized pre-TESE counselling (Krausz *et al.*, 2024).

Thanks to the application of next-generation sequencing-based WES analysis, the number of potential monogenic causes has significantly increased, paving the way for the development and implementation of gene panels (Oud *et al.*, 2025). The selection of genes for diagnostic purposes should be based on the level of GDR, as a prerequisite having at least a ‘moderate’ level. However, relatively few genes have reached this level of validation in our field, partly due to the limited number of studies and the rarity of large familial cases of infertility (Houston *et al.*, 2021). It implies that there is no standardized diagnostic gene panel for NOA patients.

Our multicentre study aimed to advance both diagnosis and TESE success prediction. Data linking monogenic factors to TESE outcomes and testis histology are extremely scarce. To address this, we analysed a virtual gene panel of 145 established monogenic causes of azoospermia in a cohort of 571 men with idiopathic NOA and known TESE outcome.

To compare our results with research papers published in the literature, our initial approach involved classifying variants according to ACMG guidelines adapting the method described by Wyrwoll *et al.* (2023). This variant classification identified 65 patients carrying LP/P variants in 40 genes, providing a plausible candidate variant in 11.3% of our cases. On the other hand, we have not identified LP/P variants in the remaining 105 genes, suggesting that variants in these genes are relatively rare causes of NOA. Over 20 studies have used gene panels to identify genetic causes of spermatogenic failure (Nakamura *et al.*, 2017; Fakhro *et al.*, 2018; Geng *et al.*, 2019a,b, 2020; Riera-Escamilla *et al.*, 2019, 2022; Araujo *et al.*, 2020; Chen *et al.*, 2020; Li *et al.*, 2020; Lorenzi *et al.*, 2020; Rocca *et al.*, 2020; An *et al.*, 2021; Cannarella *et al.*, 2021, 2023; Precone *et al.*, 2021; Wyrwoll *et al.*, 2021, 2023; Batiha *et al.*, 2022; Hou *et al.*, 2022; Kherraf *et al.*, 2022; Quarantani *et al.*, 2023; Lillepea *et al.*, 2024; Muranishi *et al.*, 2024). However, many included a small cohort of patients, analysed a limited number of genes and/or lacked ACMG-based variant classification, making it difficult to properly estimate the diagnostic yield.

Only five of these studies have screened over 10 genes in relatively large cohorts of idiopathic infertile men (>100 patients) and applied the ACMG criteria for variant classification (Chen *et al.*, 2020; An *et al.*, 2021; Wyrwoll *et al.*, 2023; Lillepea *et al.*, 2024; Muranishi *et al.*, 2024). Among these studies, diagnostic yields varied between 2.1% and 12.28%, with a strong correlation to panel size. For example, An *et al.* (2021) screened 14 genes in 668 patients and reported a diagnostic yield of 2.1%, whereas Lillepea *et al.* (2024) analysed 638 genes in 521 azoospermic/oligozoospermic men, achieving 12.28%. Despite our gene panel including ∼3.5 times fewer genes than that of Lillepea *et al.*, our diagnostic yield (11.3% by using a similar variant classification as Lillepea *et al.*, i.e. our first method) is comparable to theirs. The fact that with a lower number of genes we obtained similar results is likely to be related to the gene selection criteria in the two studies. For instance, we focused on genes exclusively related to NOA/SO, restricting the selection to those which have at least limited gene-disease (Houston *et al.*, 2021) or found to be mutated in more than one unrelated case. Conversely, Lillepea *et al.* included genes linked to broader reproductive disorders, such as hypogonadotropic hypogonadism and premature ovarian insufficiency (POI) and in genes reported in single cases of NOA. Another factor that may account for differences in diagnostic yield between studies is the proportion of patients with negative TESE outcomes. For instance, a Japanese study focusing exclusively on TESE-negative NOA patients reported a diagnostic yield of 7.83%, which is comparable to the 9.4% yield observed in our TESE-negative cohort (Muranishi *et al.*, 2024), although their diagnostic yield included AZF deletions. Finally, the differing diagnostic yields obtained using two variant classification approaches in our study population clearly suggest that this could be another explanation for discrepancies between the published studies. As a consequence of differences in variant classification practices, there is a risk that genomic datasets may sometimes be overinterpreted, leading to conclusions that might not be fully supported by the available evidence. The absence of standardized evaluation frameworks can contribute to varying interpretations and, in some cases, premature designation of genes as candidates for NOA, or to high diagnostic yield.

Although the routine diagnostic workup of NOA typically does not include the screening for monogenic causes, in some countries, diagnostic genetic laboratories have started to offer such a service. It is therefore of paramount importance to harmonize the methodology for variant classification between laboratories. The ACMG/AMP sequence variant interpretation guideline provides a framework for classifying variants based on 16 P evidence criteria and 12 B evidence criteria. However, this guideline did not elaborate on specific considerations for using evidence, the relative strength of certain evidence or for disease-specific considerations. While expert panels exist for many Mendelian disorders through ClinGen to aid genetic interpretation, no such panels currently exist for male infertility.

Here we describe a variant classification framework tailored to NOA. This method adheres to ACMG guidelines, incorporates all ClinGen recommendations and is in line with current practice in clinical genetic laboratories. The method is more conservative than the variant interpretation previously employed in male infertility literature. For instance, PM2 (variant frequency and use of control populations), which was weighted as moderate in the original ACMG/AMP guidelines, is now treated as supporting evidence due to the widespread availability of genome-wide data on allele rarity.

In addition, we applied specific thresholds for variant frequency and the use of control databases (PM2, BA1, and BS1) depending on the expected inheritance pattern (autosomal dominant, autosomal recessive, X-linked recessive, or Y-linked) and observations in healthy adults (BS2). Control databases such as gnomAD do not exclude male infertility cases, which can result in causative variants appearing at higher frequencies than in other Mendelian disorders, such as those with severe childhood phenotypes. On the other hand, variants with high allele frequency in gnomAD are unlikely to cause NOA due to the strong selective nature of this disorder, especially for autosomal variants. Therefore, we established specific thresholds based on an approximate 1% prevalence of azoospermia in the general male population and a combined genetic and allelic heterogeneity of 5% (BA1) or 1% (PM2, BS1, BS2), aligning with current knowledge on the genetic causes of infertility.

By using this approach, we identified 36 patients carrying 39 LP/P variants, resulting in a diagnostic yield of 6.1%. Although this yield may be lower compared to other Mendelian diseases, it is important to note that the field of genetic diagnosis for monogenic causes of male infertility is still in its early stages, resulting in a relatively low number of candidate NOA genes supported by published data. While the diagnostic variant classification method led to the reduction of the number of genes involved in the NOA phenotype, all the rest of our observations are concordant regardless of the classification method employed. Indeed, we observed a significant enrichment of LP/P variants in patients with testicular histology showing MA compared to those affected by SCO syndrome or HSG. Therefore, it can be expected that depending on the proportion of MA in a given study population, the diagnostic yield may be higher if the MA phenotype is more prevalent. A plausible explanation for this observation is that the genetic background of MA seems to be primarily associated with mutations in a limited number of genes crucial for meiosis, whereas SCO and HSG are likely linked to a more heterogeneous genetic landscape. Moreover, we can speculate that for less severe infertile phenotypes, such as HSG or oligozoospermia, environmental factors may play a more significant role than in SCO or MA.

We found a strong association between carrying an LP/P variant and failure to retrieve spermatozoa using TESE, consistent across the different participating laboratories (Barcelona, Doha, and Newcastle/Nijmegen), and when combining our data with literature. While Wyrwoll *et al.* (2023) reported nearly no success in TESE success for men with genetically diagnosed NOA, our study shows that sperm retrieval success is gene dependent. Variants in 19 genes were consistently linked to negative TESE outcomes, with genes such as *MSH4, SYCE1*, and *TEX11* mutated in a relatively large number of patients. Therefore, patients carrying LP/P variants in these genes should be informed that the likelihood of obtaining spermatozoa by TESE is virtually zero. Conversely, our work highlights that mutations in the other 11 genes might be occasionally compatible with sperm production with a variable positive sperm retrieval success rate. For example, TESE was successful in half of the patients with LP/P variants in the *M1AP* and *ADAD2* genes, while such variants in *AR* were associated with an ∼80% failure rate, though the number of patients with LP/P in *AR* and *ADAD2* remains low. Notably, in 6 of the 11 genes in cases where sperm was recovered, LP/P variants are X-linked, meaning they will be obligatorily transmitted to daughters if ICSI succeeds, increasing the risk of propagating NOA in the grandsons. Therefore, patients carrying variants in these genes should be counselled about the feasibility of TESE, and the consequences for the descendants. Moreover, further understanding of the relationship between the affected gene and ICSI success is crucial, though no data currently exists on ICSI outcomes in monogenic forms of NOA. We have examined potential biological differences between genes linked to negative TESE outcomes and those compatible with sperm production by analysing their function, expression, and reproductive phenotypes in mice. Both gene groups are enriched for meiosis-associated genes, but mRNA expression patterns revealed subtle differences. Genes associated with negative TESE outcomes have a more prevalent mRNA expression in spermatogonia and spermatocytes, whereas genes associated with positive TESE outcomes show a more heterogeneous expression, including detection in somatic cells and across early and late spermatogenic stages. This suggests that genes associated with negative TESE outcomes may have a more confined role in early spermatogenic stages, while those compatible with sperm production may function across various phases of germ cell maturation. Although mice may not represent a perfect model for human NOA since they produce 10 times more spermatids per gram of testis compared to humans (Fayomi and Orwig, 2018), many KO mice for genes associated with negative TESE outcomes fail to produce mature spermatids, reinforcing their crucial role in mammal spermatogenesis. A particularly intriguing subset of genes are those that exhibit both negative and positive TESE results. Future research should explore whether factors such as an individual’s genetic background, age, or environmental influences may contribute to the heterogeneous TESE phenotype.

A wider implication of our data concerns female infertility. In a previous WES study on MA patients, we suggested that some of the MA-associated genes could also contribute to POI (Krausz *et al.*, 2020). In the current cohort, nine genes with LP/P have already been linked to POI with at least a limited GDR (Van Der Kelen *et al.*, 2023; *FANCA, MCM9, MEI1, MEIOB, MSH4, PSMC3IP, STAG3, SYCE1*, and *UBR2*). Interestingly, with the exception of *UBR2*, where mutations are compatible with sperm production in the testis, the other eight genes have been associated with negative TESE outcomes. Although it is not known whether the remaining genes may lead to female infertility, it is noteworthy that in seven of them (*ADAD2, AR, C1orf146, MLH3, RAD21L1, SYCP3*, and *TERB1)*, the corresponding female KO mice are infertile. We therefore propose these genes as potential novel candidates for POI. The identification of LP/P variants in established or candidate POI genes implies that genetic counselling for NOA has relevance not only to the male but also to the female family members. In younger female siblings, if presenting the same genotype, fertility preservation could be advised prior to ovarian failure.

Some limitations of this study are inherent to the genetic architecture of NOA. Azoospermia is highly heterogeneous from a genetic perspective, with hundreds of candidate NOA genes reported in the literature (Riera-Escamilla and Nagirnaja, 2025).

It has been estimated that the number of azoospermia genes can be close to 600 genes (Nagirnaja *et al.*, 2022). The relatively low number of genes (n = 145) used in this study may be considered as a limitation, however, as stated above, our aim was to evaluate only those NOA genes which have clear evidence from the available literature. Indeed, our gene panel includes only recurrently mutated genes which reached at least limited evidence for GDR in relationship with NOA. Although our study represents the largest population to date with known TESE outcomes, we were unable to draw definitive conclusions, particularly regarding the prediction of negative TESE outcomes. Based on both our findings and the currently available data, it is unlikely to find multiple unrelated cases of NOA caused by mutations in the same gene. As already pointed out by Nagirnaja *et al.* (2022), the Mendelian causes of NOA are broadly distributed across a variety of genes, rather than being clustered within a small number of specific genes (Nagirnaja *et al.*, 2022). For this reason, the number of mutation carriers for each gene is low even when combined with literature data. In addition, only a minority of the published papers contain information on TESE success. While the low number of carriers of genes associated with positive sperm retrieval does not limit our conclusions about TESE prediction, caution should be exercised when interpreting genes linked to negative TESE outcomes. To date, only for three genes, *TEX11*, *SYCE1*, and *MSH4*, each of which has 10 or more reported cases, we can confidently state that the likelihood of finding spermatozoa is extremely low. For the remaining TESE-negative genes, we need more data in order to provide stronger evidence for the observed genotype–phenotype correlations.

The use of different TESE approaches in different clinics may account for those cases where LP/P variants in the same gene were associated with both TESE-positive and TESE-negative outcomes. This potential limitation concerns all published studies analysing genetic factors versus TESE outcomes. However, such a limitation should be considered minimal, since Corona *et al.* (2019) demonstrated that there are no significant differences in sperm retrieval rates between cTESE and micro-TESE in men with NOA.

Another potential limitation concerns the way how each laboratory apply the ACMG/AMP guidelines for interpreting variant pathogenicity. To ensure transparency, we have explicitly stated the scores assigned to each parameter in our evaluations. Moreover, we classified variants by using two different interpretation criteria: one adapted from the method previously reported by Wyrwoll *et al.* (2023), to allow comparison with results published in the literature, and another used in diagnostic laboratories for Mendelian diseases. The latter, applied for the first time in the literature, aims to reduce false positives. While applying the ACMG guidelines, variants classified as LP or P have an estimated >90% and >99% likelihood, respectively, of being truly P. Indeed, in the clinical practice, these variants are reported for diagnostic purposes. However, in some instances, functional validation, especially for missense variants, would provide definitive evidence of pathogenicity. We have been very conservative in our classifications, so any variant with such uncertainties was instead classified as a VUS. We acknowledge the need for broader consensus among experts and hope for the establishment of a male infertility ClinGen gene expert panel, as well as a standardized approach to variant classification in the field of androgenetics.

## Conclusion

Here, we provide the genetic diagnosis in at least 6.1% of men previously classified as affected by idiopathic azoospermia. The relatively high number of subjects with LP/P variants in X chromosome-linked genes further underscores the relevance of this sex chromosome in male reproductive fitness and highlights the importance of including these genes in future gene panels (Riera-Escamilla *et al.*, 2022). X chromosome gene defects will be obligatorily transmitted to female offspring, carrying the potential to propagate NOA to male descendants in subsequent generations. Our study clearly indicates that patients with germ cell arrest have the highest probability of being affected by monogenic defects and are most likely to have a negative TESE outcome. Thanks to our study, we were able to ‘upgrade’ the GDR for 26 genes, and 35 have now reached ‘moderate’ or higher clinical evidence. This implies that they could be already integrated in a routine, diagnostic gene panel addressing NOA men. Such a comprehensive approach could significantly enhance our ability to determine the aetiology and improve pre-TESE counselling. The identification of genes associated with potential sperm production in the testis and of genes that have been consistently mutated in patients with negative TESE outcomes represents a substantial step forward in precision medicine, offering more accurate genetic counselling and informed decision-making to the patients.

## Supplementary Material

hoaf049_Supplementary_Data

## Data Availability

The data underlying this article will be shared on reasonable request to the corresponding authors. LP/P variants identified in our cohort have been submitted to ClinVar (https://www.ncbi.nlm.nih.gov/clinvar/).
