# Supplementary material for: Genetic determinants of testicular sperm extraction outcomes: insights from a large multicentre study of men with non-obstructive azoospermia
Source: Hum Reprod Open. 2025 Aug 29;2025(3):hoaf049. doi: 10.1093/hropen/hoaf049 (PMC12396851; doi:10.1093/hropen/hoaf049)

**Supplementary File S1 – IGV plots**

For seven LP/P variants DNA was not available for Sanger sequencing and we examined the quality of sequencing reads through the Integrative Genomics Viewer (IGV)

1. WES_INF_187, *NANOS2*, NM_001029861.3:c.3G>A, Homozygosis


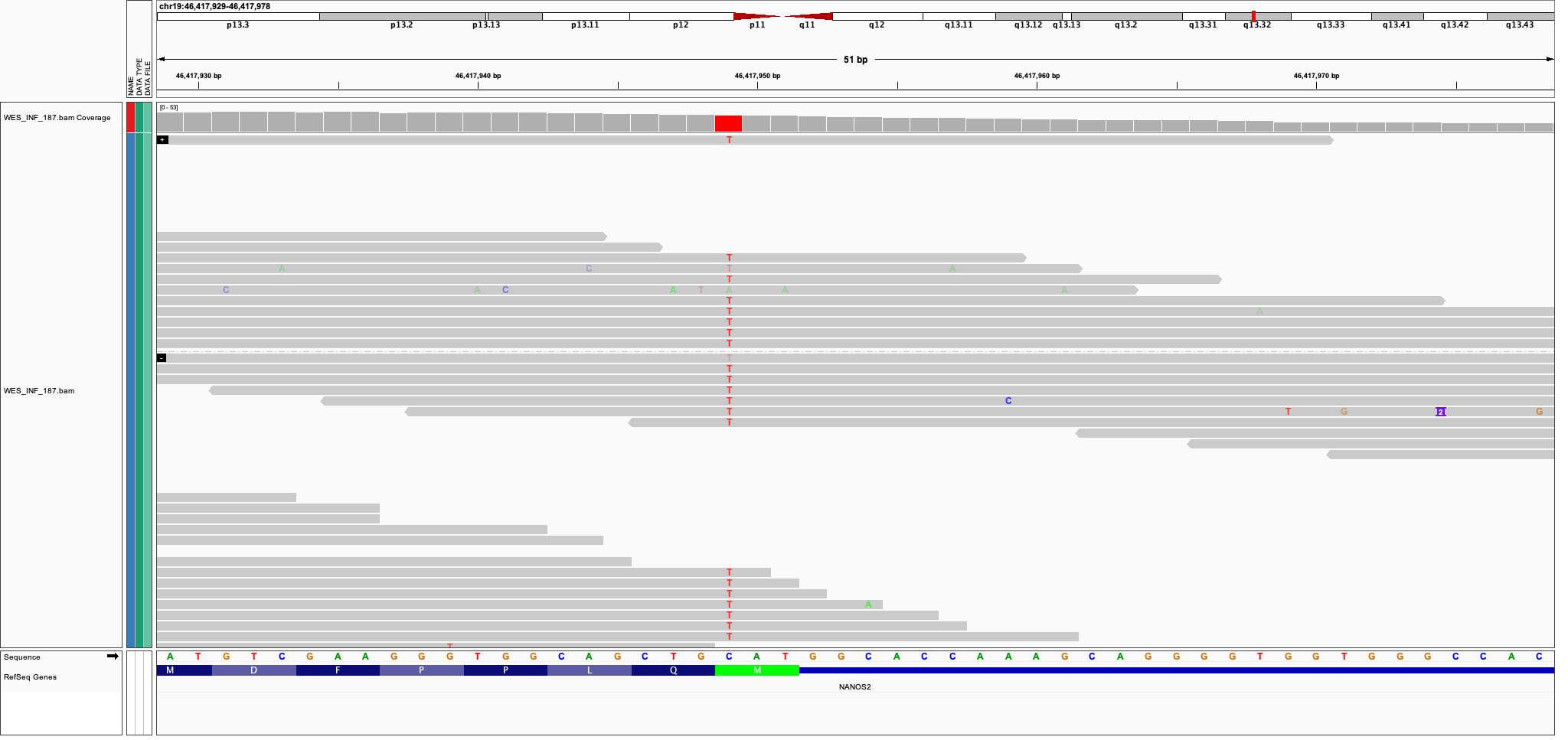


2. WES_INF_284, *MSH4*, NM_002440.4:c.2698C>T, Homozygosis


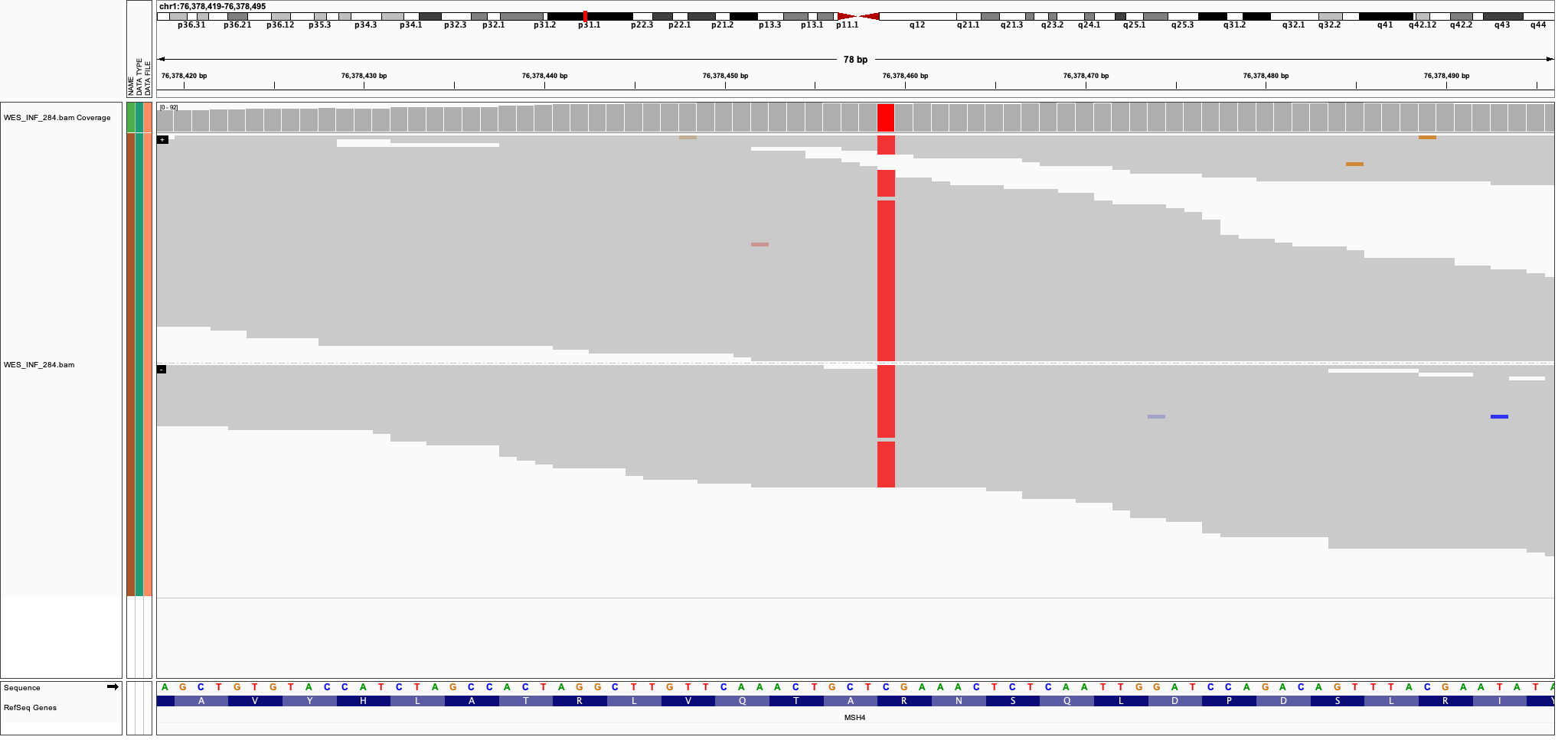


3. WGS_INF_127, *SPO11*, NM_012444.3:c.556G>A, Homozygosis


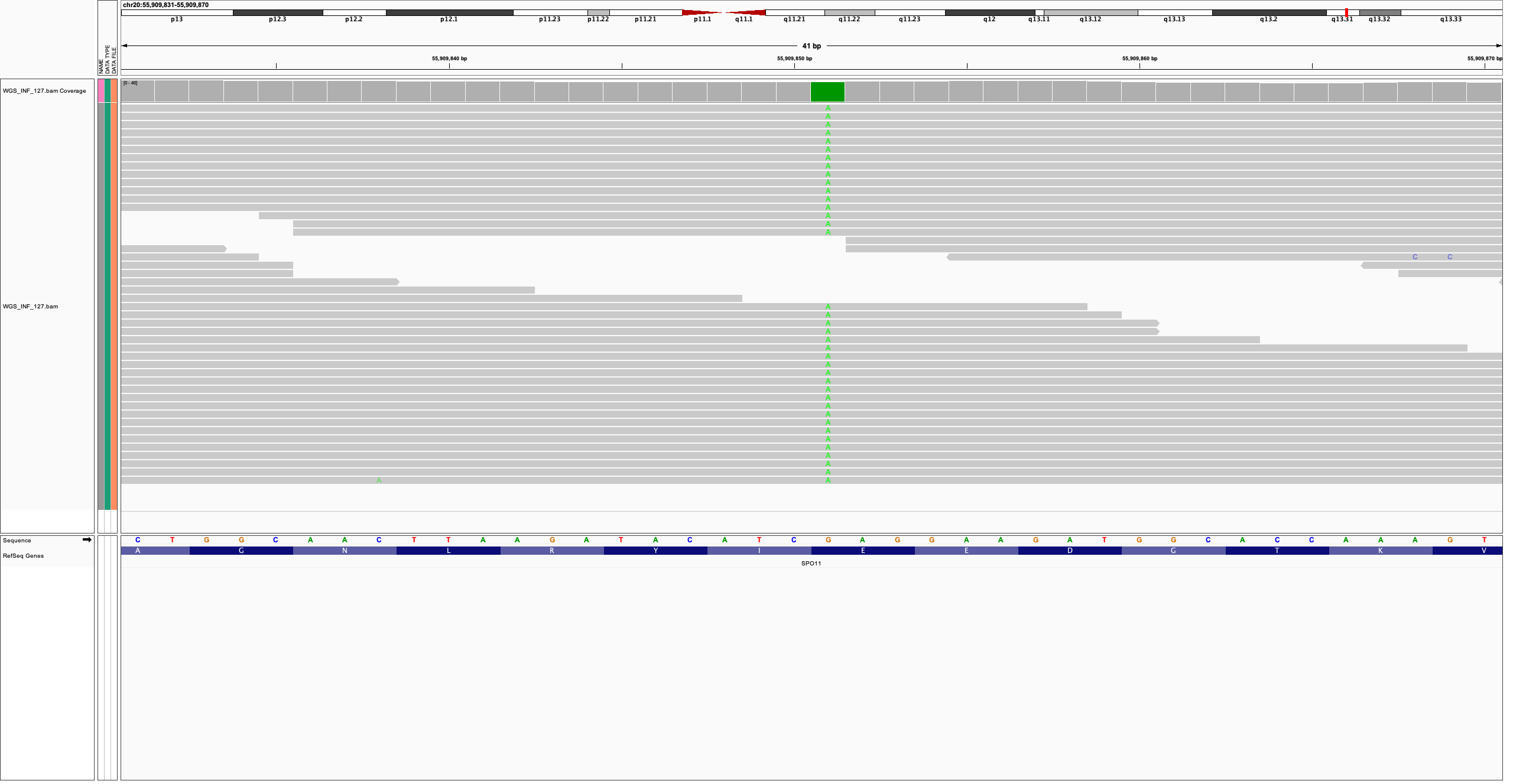


.

4. WGS_INF_154, *RBBP7*, NM_002893.4:c.503C>G, Hemizygosis


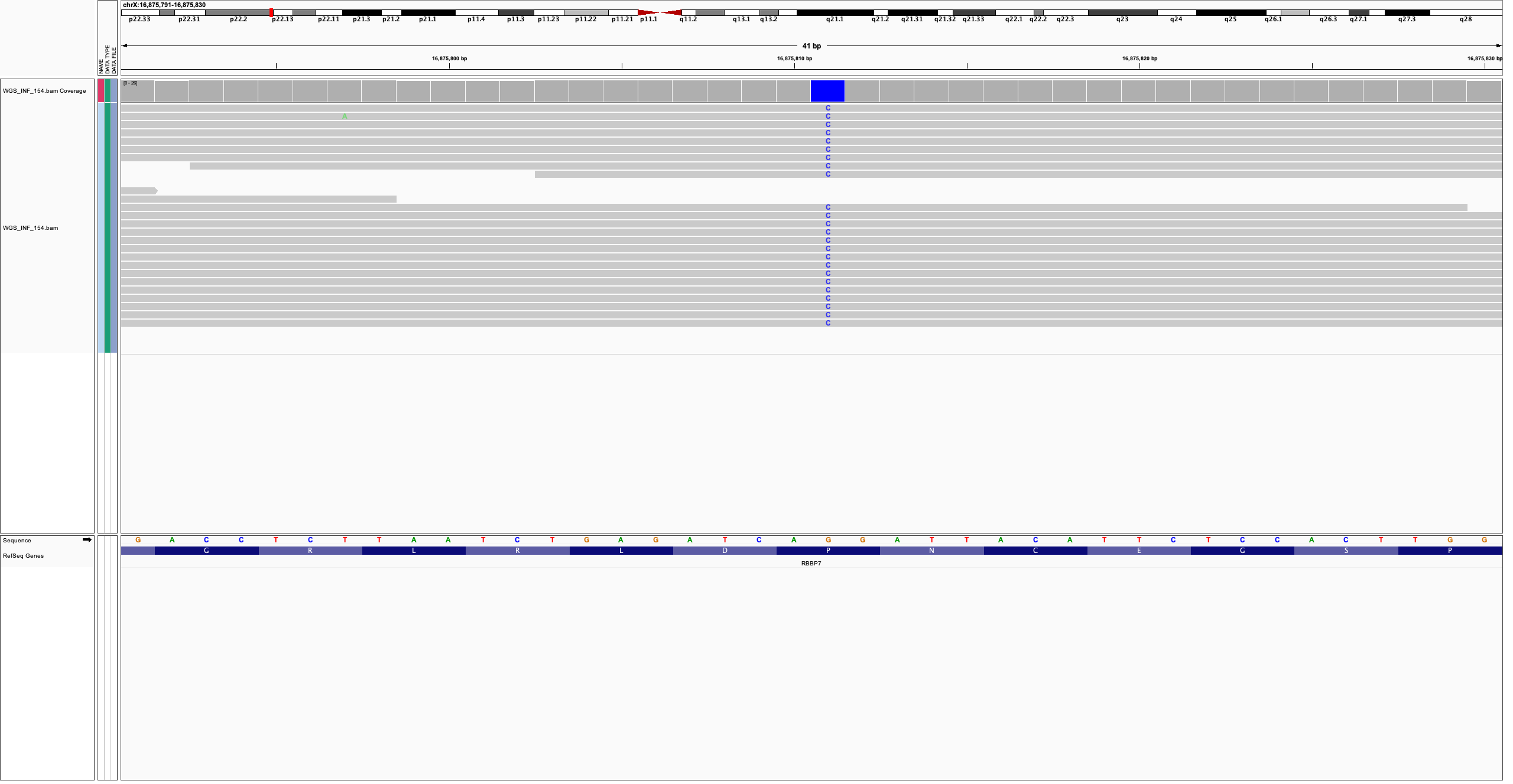


5. WGS_INF_160, *PSMC3IP*, NM_016556.4:c.617C>T, Homozygosis


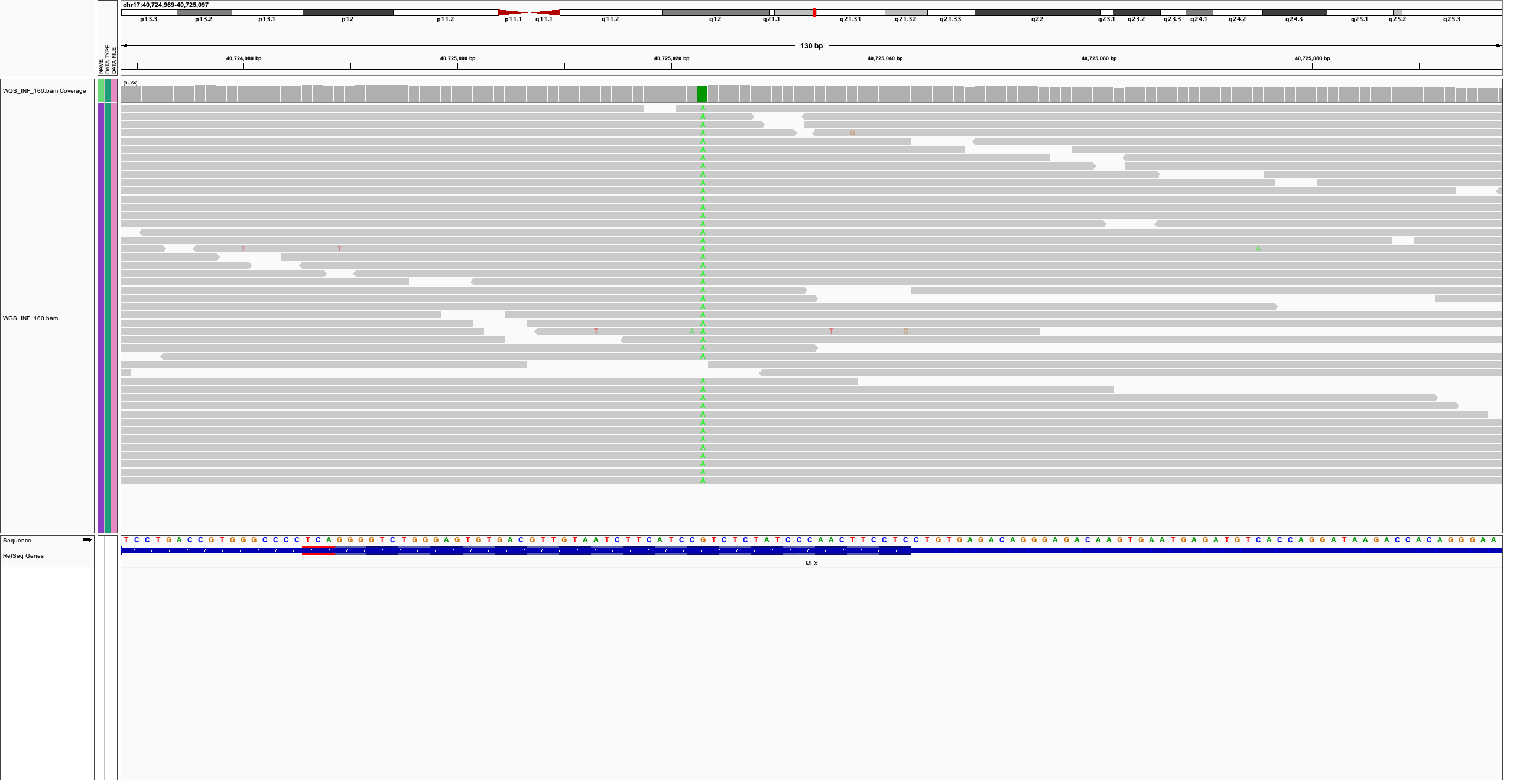


6. NCL_MI_0008P, *STAG3*, NM_001282717.2:c.130_131del, Homozygosis


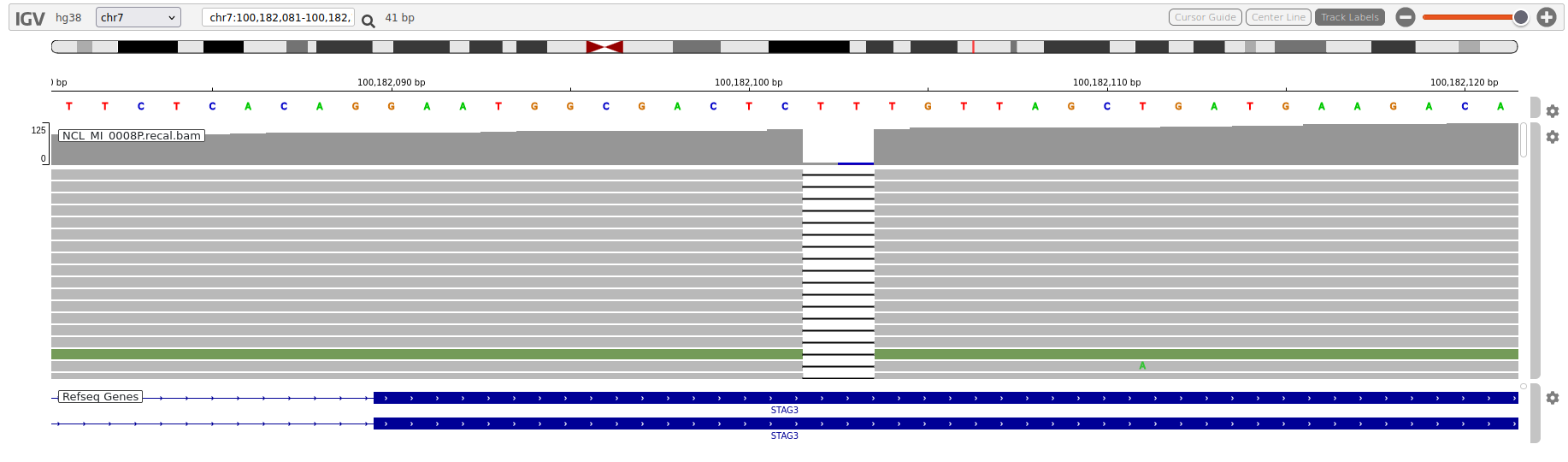


7. NCL_MI_0121P, *UBR2*, NM_001363705.2:c.345_346del, Heterozygosis


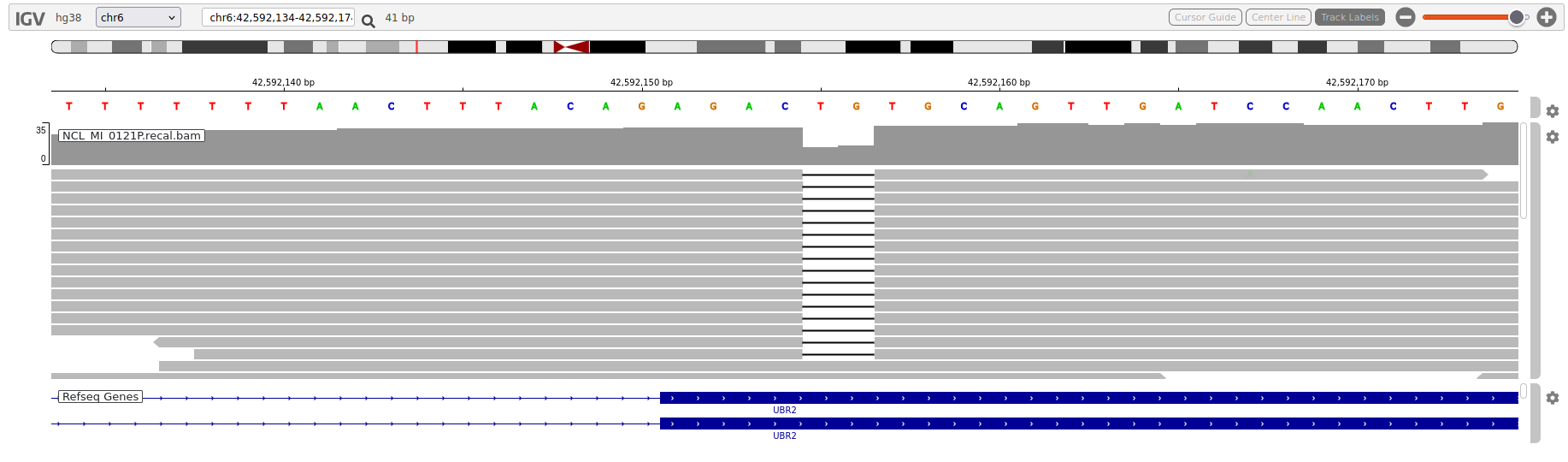

Supplement: hoaf049_Supplementary_Data [file hoaf049_supplementary_data.zip › HRO-25-0045.R2_Supplementary_file_S1.docx]
