# Supplementary material for: Genetic determinants of testicular sperm extraction outcomes: insights from a large multicentre study of men with non-obstructive azoospermia
Source: Hum Reprod Open. 2025 Aug 29;2025(3):hoaf049. doi: 10.1093/hropen/hoaf049 (PMC12396851; doi:10.1093/hropen/hoaf049)
Supplement: hoaf049_Supplementary_Data [file hoaf049_supplementary_data.zip › HRO-25-0045-R2-SuppData.docx]

**Supplementary Data**

**Genetic Determinants of Testicular Sperm Extraction Outcomes: Insights from a Large Multicenter Study of Men with Non-Obstructive Azoospermia**

Antoni Riera-Escamilla, Mohamed M Arafa, Ginevra Farnetani, Miguel J. Xavier, Manon S. Oud, Ahmad A. Majzoub, Liliana Ramos, Chiara Abrardo, Matilde Spinelli, Daniel Moreno-Mendoza, Giuseppe Defazio, Elisabet Ars, Marc Pybus, Josvany R Sánchez Curbelo, Haitham T Elbardisi, Shoaib Nawaz, Najeeb Syed, Eduard Ruiz-Castané, Godfried W. van der Heijden, Khalid A. Fakhro, Joris A. Veltman, Csilla Krausz

**Contents**

**Supplementary Materials and Methods**

**Supplementary Figure S1** – Schematic representation of the study design

**Supplementary Figure S2** – Network of biological processes affected by genes with LP/P variants identified using the ACMG/AMP adapted from Wyrwoll et al. (2023) (Panel A) and ACMG/AMP-based variant interpretation framework refined to genetic diagnostics in non-obstructive azoospermia (Panel B) linked to positive TESE outcomes. Redundant GO terms are collapsed into common biological themes. Each node represents a term affected by at least three genes in the disease-linked gene set.

**Supplementary Figure S3** – Network of biological processes affected by genes with LP/P variants identified using the ACMG/AMP adapted from Wyrwoll et al. (2023) (Panel A) and ACMG/AMP-based variant interpretation framework refined to genetic diagnostics in non-obstructive azoospermia (Panel B) linked to negative TESE outcomes. Redundant GO terms are collapsed into common biological themes. Each node represents a term affected by at least three genes in the disease-linked gene set.

**Supplementary Table S1** – Summary of the sperm parameters from the normozoospermic control cohort.

**Supplementary Table S5** – Summary of the comparisons performed on carriers of VUS and LB/B variants according to two different ACMG-AMP variant classification methods. Comparisons were performed in function of TESE outcome.

**Provided as separate files:**

**Supplementary Table S2** – List of 145 genes linked to non-obstructive azoospermia or severe oligozoospermia screened in our cohort

**Supplementary Table S3** – Clinical, genetic and histological parameters and TESE outcomes of the 571 patients sequenced in this study

**Supplementary Table S4** – Rare non-synonymous exonic and splicing variants identified in a cohort of 571 azoospermic patients, classified using ACMG/ClinGen criteria (both methods)

**Supplementary Table S6** – Rare non-synonymous exonic and splicing variants reported in the literature, classified using ACMG/ClinGen criteria described in Wyrwoll et al. (2023)

**Supplementary Table S7** – LP/P variants classified by ACMG/ClinGen criteria with less stringent thresholds in patients with known TESE outcomes, reclassified using the diagnostic method

**Supplementary Table S8** – TESE outcomes, testis expression and KO mouse model data for 40 genes with LP/P variants classified using the criteria employed by Wyrwoll et al. (2023)

**Supplementary Table S9** – TESE outcomes for genes with LP/P variants classified using the diagnostic criteria

**Supplementary File S1 – IGV plots**

**Supplementary Materials and Methods**

**Exome and Genome sequencing**

**Barcelona cohort:**

All patients underwent exome sequencing (WES). WES samples were prepared and enriched following the manufacturer’s protocols of SureSelectV6 from Agilent Technologies. All sequencing was performed on the NovaSeq 6000 Sequencing System (Illumina) at an average depth of >100X. The average depth for the exonic regions belonging to the gene panel was 160.8 (average), 158.5 (median), and 145-176 (range) being the coverage >20 in >94% of all regions. The bioinformatics analysis was performed using the Illumina pipeline and consisted of nucleotide assignment and demultiplexing of sequenced fragments, grouping them according to the patient of origin. The quality of the reads was verified using FastQC (<http://www.bioinformatics.babraham.ac.uk/projects/fastqc/>). The sequencing fragments were aligned to the hg19 reference genome using the Burrows-Wheeler aligner (bwa-mem) (<https://github.com/lh3/bwa>). Samtools was used to sort and mark duplicated mapped reads (<http://www.htslib.org/>). To prevent the propagation of PCR artifacts, PCR duplicates were removed using Picard. GATK was used to realign complex regions—potential InDels and SNP-rich regions—as well as to recalibrate nucleotide quality once the entire alignment process was completed. The resulting alignments were analyzed by the GATK HaplotypeCaller program to identify SNVs and InDels

**Newcastle/Nijmegen cohort:**

As previously described in Oud et al. (2022)(Oud *et al.*, 2022), 156 WES samples were prepared and enriched following the manufacturer’s protocols for either Illumina’s Nextera DNA Exome Capture Kit or Twist Bioscience’s Twist Human Core Exome Kit. All sequencing was performed on the NovaSeq 6000 Sequencing System (Illumina) at an average depth of 94X (Median: 85X, Range: 41.1X–239X). Sequenced reads were aligned to the human reference genome (GRCh38/hg38) using BWA-MEM v0.7.17(https://github.com/lh3/bwa), Picard (https://broadinstitute.github.io/picard/), and GATK v4.1.4.1(https://github.com/broadinstitute/gatk/). Following best practice recommendations, single nucleotide variants (SNVs) and small indels were identified and quality-filtered using GATK’s HaplotypeCaller(Poplin *et al.*, 2017), with variants having GQ ≥ 20 classified as high-confidence.

The 43 WGS samples were analyzed using the same pipeline as WES, except that the Illumina DNA PCR-Free Prep for Human Whole Genome Kit was used for library preparation, and Spark was employed instead of Picard. The average coverage for WGS samples was 43X (Median: 34.2X, Range: 25X–118X).

For the exonic regions belonging to the gene panel, WES had an average depth of 70X (Median: 75X, Range: 36X–126X), while WGS had an average depth of 35X (Median: 36X, Range: 23X–61X).

**Doha cohort:**

WGS samples were processed by Illumina HiseqX machines at Sidra Medicine. Quality control (QC) metrics were assessed at various stages to ensure data accuracy. FastQC (version 0.11.2) was used to evaluate raw sequencing data, while a combination of SAMtools(https://www.htslib.org/), Picard and mosdepth (https://github.com/brentp/mosdepth) were employed for QC analysis of mapped reads. To verify pedigree information, Peddy (<https://github.com/brentp/peddy>) was run independently for each families to infer familial relationships from the genotyped data. In brief, sequencing was performed using the Illumina HiSeq X platform, generating 150 bp paired-end reads. Reads were aligned to the GRCh37/hg19 reference genome using BWA (version 0.7.10)(Li and Durbin, 2009). Sequence-level variants were identified using GATK (version 4.1) following the best practices pipeline. The average depth for the exonic regions belonging to the genes was 36 (average), 37 (median), and 23-53 (range), being the coverage >20 in >96% of all regions.

**Variant filtering**

In order to obtain a list of variants annotated with the same transcript and, therefore, the same predicted consequences and MAF, variants were lifted over to hg38 (when needed) and annotated using Ensembl Variant Effect Predictor (VEP) release 112(McLaren *et al.*, 2016)

**CNV detection**

**Barcelona cohort:**

ExomeDepth was used to detect CNVs with the default pipeline configuration. The reference set for each sample was constructed from the remaining samples in the cohort. Duplications were removed, and deletions with a frequency >1% in gnomAD SV4.1.0 or found in >10 cases were excluded. All candidate deletions were manually inspected using IGV, and those deemed reliable were validated through qPCR or +/-PCR.

**Newcastle/Nijmegen cohort:**

For WGS trio data analyzed using CNVRobot (<https://github.com/AnetaMikulasova/CNVRobot>) and dysgu-sv (https://github.com/kcleal/dysgu), we filtered out GAINs/DUPs, CNVs labeled as high noise or low mappability, small CNVs (<2kb) detected only by dysgu-sv, CNVs with high population frequency (>0.01) or recurrent in the cohort (>10 cases), and paternally inherited CNVs. The remaining deletions were visually inspected through IGV. For WES singleton data we used CNVRobot applying similar filtering criteria, removing GAINs/DUPs, high-noise CNVs, and those with high population frequency.

**Doha cohort:**

For structural variants analysis, we employed three structural variant callers: Delly version 0.7.8 (Rausch et al. 2012(Rausch *et al.*, 2012)), Speedseq version 0.1.2 (Chiang et al. 2015(Chen *et al.*, 2016)), and Manta version 1.6.0 (Chen et al. 2016), and applied the best practices recommended for each tool. We retained only SVs reported by at least two tools, with sizes ranging from 50 bp to 10 Mb. The annotation of structural variants was carried out using ANNOTSV version 2.2(Geoffroy *et al.*, 2023)

**Variant Classification (method I, adapted from Wyrwoll et al. 2023)**

Similar to Wyrwoll et al. (2023)(Wyrwoll *et al.*, 2023) we classified all variants according to the ACMG/Clingen criteria based on the following parameters:

**PVS1**: criteria were applied as very strong criteria only to LoF variants, including (partial and complete) gene deletions in genes where LoF variants had already been described for male infertility, removed >10% of the protein, and were not located in the last exon. It was downgraded to strong according to Tayoun et al. 2018(Abou Tayoun *et al.*, 2018) if the LoF variant occurred in the last exon of the gene and the variant removed >10%. We classified variants as moderate if they were in the last exon and removed <10% of the protein. **PS1**: we classified variants as strong when the same amino acid change was previously established pathogenic variant regardless of nucleotide change. **PS2**: None of the variants reported in this study were proven to be de novo. **PS3**: We classified it as moderate when two independent assays or one assay with very convincing data demonstrated the pathogenicity of the variant and as supporting when only one assay supported a functional effect (Brnich *et al.*, 2019). **PS4**: According to Harrison et al. (2019)(Harrison *et al.*, 2019), we considered a variant as moderate when it was found in at least two unrelated cases described in the literature, a disease-specific database, or an in-house cohort showing a similar phenotype. It was downgraded to supporting whether the same variant was observed in only one independent individual. **PM1**: we used Metadome (https://stuart.radboudumc.nl/metadome/) to analyse if a variant occurs in a functional protein domain and whether the affected amino acid residue is within an intolerant region. In particular, we applied moderate if a variant was located in an intolerant region belonging to a known functional protein domain, and it was downgraded to supporting when the amino acid residue was within an intolerant protein region but outside any known protein domain. **PM2** was applied moderate to variants with a gnomAD MAF<0.01 for genes with a recessive mode of inheritance and <0.001 for X-, Y-linked, or dominant genes. **PM3**: As recommended by the ClinGen working group (https://clinicalgenome.org/site/assets/files/3717/svi_proposal_for_pm3_criterion), we classified them as moderate variants, for which we were able to demonstrate that they were in trans. The homozygous variants were downgraded for supporting. **PM4**: Moderate was applied when >1 amino acid was inserted or deleted in a non-repeated region. It was downgraded to supporting if only one amino acid had been inserted or deleted. **PM5**: we classified as supporting if one additional substitution for the respective amino acid residue has been observed and upgraded to strong when two or more different amino acid substitution in addition to the observed amino acid substitution were described in the literature or databases. **PP1**: according to Jarvik and Browning recommendations(Jarvik and Browning, 2016), all informative meiosis of a given variant were counted when phenotype and genotype were provided. For recessive genes, at least two informative meiosis events need to be observed in order to apply supporting. **PP2**: was applied to missense variants located in genes with observed/expected ratios of missense variants (o/e ratio) <0.65 according to gnomAD v4.1 (gnomAD v2.1.1 for X-linked genes). **PP3**: was applied supporting in missense variants with an in-house index of pathogenicity ≥0.7, based on six prediction tools, and was calculated as described by Krausz et al. 2020(Krausz *et al.*, 2020). **PP4**: supporting was used if the patient’s testicular phenotype was highly specific to the gene. **PP5**: was not applied as recommended by Biesecker and Harrison, 2018(Biesecker *et al.*, 2018).

**BS1**: does not apply since we already filtered variants according to their MAF. **BS2**: was considered very strong if the same genotype was found in a normozoospermic fertile control from our in-house database. **BS3**: We applied moderate when two independent assays or one assay with convincing data demonstrated that the variant had no functional effect. **BS4**: none of the variants fits this category “Lack of segregation in affected members of a family”. **BP1**: was applied for missense variants in the case of a gene for which only truncating variants were reported to lead to male infertility. **BP2**: in our cohort, none of the identified variants were observed in trans with a pathogenic variant for a fully penetrant dominant gene/disorder or in cis with a pathogenic variant in any inheritance pattern. **BP3**: we applied supporting when an In-frame deletions/insertions was located in a repetitive region without a known function.**BP4**: we applied supporting those variants with an in-house index of pathogenicity <0.5. **BP5**: supporting was applied if a further (likely) pathogenic variant was observed in the proband. **BP6**: We applied supporting for variants in which ClinVar (<https://www.ncbi.nlm.nih.gov/clinvar/>) and/or LOVD (https://www.lovd.nl/) unanimously classified them as benign or likely benign.

**Variant Classification applying all ClinGen recommendations (method II)**

**PVS1:**

- **Very Strong**: LoF is a mechanism of disease. The variant is a nonsense, frameshift, canonical splice site (spliceAI >= 0.9) or deletion affecting an exon in a biologically active transcript, or a duplication proven in tandem. The variant is predicted to cause NMD (variant not located in last or last 50bp of penultimate exon).

- **Strong**: The variant is a nonsense, frameshift, canonical splice site (spliceAI >= 0.9) or deletion not predicted to cause NMD, but affecting a region that is crucial to the transcript. Alternatively the role of region in protein function is unknown, but the variant affects an exon in a biologically active transcript AND removes >10% of the protein.

- **Moderate:** The variant is a nonsense, frameshift, canonical splice site (spliceAI >= 0.4) or deletion not predicted to cause NMD, the role of region in protein function is unknown, but the variant affects an exon in a biologically active transcript AND removes <10% of the protein OR an initiation codon is disrupted, there are no known other start codons in other transcripts and there is >= 1 pathogenic variant(s) upstream of closest potential in-frame start codon.

- **Supporting**: Initiation codon is disrupted, there are no known other start codons in other transcripts and there are no pathogenic variants upstream of closest potential in-frame start codon

**PS1:**

**- Strong**: Same amino acid change was previously established pathogenic variant regardless of nucleotide change (does not apply together with PM4)

**PS2:**

- **Very Strong:** Two or more identical variants proven to be de novo (paternity and maternity confirmed) AND the phenotype is highly specific for the gene.

- **Strong**: Variant proven to be de novo (paternity and maternity confirmed) AND the phenotype is highly specific for the gene.

- **Moderate:** Variant proven to be de novo (paternity and maternity confirmed) AND the phenotype is consistent with gene, but not highly specific OR Variant appears to be de novo (paternity and maternity not confirmed) AND the phenotype is highly specific for the gene.

- **Supporting:** Variant appears to be de novo (paternity and maternity not confirmed) AND the phenotype is consistent with gene, but not highly specific.

**PS3:**

- **Very Strong**: The assay is applicable for the variant/gene/disease AND OddsPath >350.

- **Strong**: The assay is applicable for the variant/gene/disease AND OddsPath >18.7.

- **Moderate**: The assay is applicable for the variant/gene/disease AND at least 11 pathogenic/benign control variants were included in the assay

- **Supporting**: The assay is applicable for the variant/gene/disease AND 10 or less variant controls were included in the assay.

**PS4:**

- **Strong**: >= 15 unrelated patients with consistent phenotype AND meeting the PM2 criterium.

- **Moderate**: >= 6 unrelated patients with consistent phenotype AND meeting the PM2 criterium.

- **Supporting**: >= 2 unrelated patients with consistent phenotype AND meeting the PM2 criterium.

**PM1:**

- **Moderate**: Located in a mutational hot spot and/or critical and well-established functional domain (e.g. active site of enzyme) without benign variation

at a minimum, one or more of the following should be apparent:

● Evidence of local enrichment of pathogenic missense variation

● Evidence from protein or protein domain paralogs of pathogenic variation at the paralogous residue

● Evidence that the residue lies in an invariant position in a functionally well-established domain (e.g.

enzyme active site)

● Evidence from in silico protein modelling studies predicting a likely deleterious structural or ligandbinding impact in a region of the protein with a known function

- **Supporting**: Located in a mutational hot spot and/or critical and well-established functional domain (e.g. active site of enzyme) without benign variation.

**PM2:**

- **Supporting**: using gnomAD v.4: Recessive: MAF ≤ 0.01 (1%); Dominant: MAF ≤ 0.00005; X-linked: MAF ≤ 0.0001; Y-linked: MAF ≤ 0.0001

**PM3:**

- **Very Strong**: Variant demonstrated to be in trans with another (likely) pathogenic variant in 4 unrelated probands.

- **Strong**: Variant demonstrated to be in trans with another (likely) pathogenic variant in 2 unrelated probands.

- **Moderate**: Variant demonstrated to be in trans with another (likely) pathogenic variant in 1 proband OR homozygous occurence of a VUS in at least 2 unrelated probands.

- **Supporting**: Homozygous occurence of a VUS or proved compound heterozygous

**PM4:**

- **Moderate:** > 1 amino acid inserted or deleted in a non-repeated region.

- **Supporting**: 1 amino acid inserted or deleted in a non-repeated region.

**PM5:**

- **Moderate**: Previously identified missense variant is classified as pathogenic and is identified in >=2 unrelated probands. The variant being assessed must have a similar or greater predicted impact on the protein than the reference variant (assessed using REVEL score)

- **Supporting**: Previously identified missense variant is classified as (likely) pathogenic and is only identified in 1 proband. The variant being assessed should have a similar or greater predicted impact on the protein than the reference variant (assessed using REVEL score)

**PM6**: Does not apply

**PP1:**

- **Strong**: Variant segregates with >= 7 meiosis

- **Moderate**: Variant segregates with >= 5 meiosis

- **Supporting**: Variant segregates with >= 3 meiosis

**PP2:**

- **Supporting** Missense variants located in genes with observed/expected ratios of missense variants (Z-score >= 3.09 ) according to gnomAD v4.1.0 AND position has an missense observed/expected ratio of <0.2 OR located in a highly intolerant region (dn/ds <0.2) according to Metadome. Does not apply if PM1 applied

**PP3:**

- **Strong**: REVEL score >=0.932.

- **Moderate**: REVEL score 0.773-0.932

- **Supporting**: REVEL score 0.644-0.773

**PP4:**

- **Very Strong**: The diagnostic yield is more than 97.5% for the specific phenotype.

- **Strong**: The diagnostic yield is more than 68.0% for the specific phenotype

- **Moderate**: The diagnostic yield of the gene is more than 33% for the specific phenotype.

- **Supporting**: The diagnostic yield of the gene is more than 20% for the specific phenotype

**PP5:** does not apply

**BA1**:

- **Very Strong**: if allele frequency (AF) in gnomAD v.4: Autosomal recessive: AF >0.02236;

Autosomal dominant: AF > 0.00025, X-linked: AF >0.0005; Y-linked: AF >0.0005

**BS1**:

- **Strong**: if allele frequency (AF) in gnomAD v.4: Autosomal recessive: AF 0.01- 0.02236; Autosomal dominant: AF 0.00005 - 0.00025; X-linked: AF 0.0001 – 0.0005; Y-linked: AF 0.0001 – 0.0005

**BS2:**

- **Very Strong**: Same genotype observed in a normozoospermic control from our in-house database.

- **Moderate**: Same genotype identified in >40 XY individuals in gnomAD

**BS3**:

- **Strong**: The assay is applicable for the variant/gene/disease AND OddsPath <0.053

- **Moderate**: The assay is applicable for the variant/gene/disease AND at least 11 pathogenic/benign control variants were included in the assay.

- **Supporting**: The assay is applicable for the variant/gene/disease AND 10 or less variant controls were included in the assay.

**BP1**:

- **Supporting**: Missense variants in the case of a gene for which only truncating variants were reported to lead to male infertility. The gene need to have a gene-disease relationship of Definitive evidence

**BP2**

- **Supporting**: observed in trans with a pathogenic variant for a fully penetrant dominant gene/disorder or in cis with a pathogenic variant in any inheritance pattern.

**BP3:**

- **Supporting**: In-frame deletions/insertions located in a repetitive region

**BP4**:

- **Very Strong**: REVEL score <=0.003

- **Strong**: REVEL score 0.003-0.016

- **Moderate**: REVEL score 0.016-0.183

- **Supporting**: REVEL score 0.183-0.29. For splicing variants, Splice AI delta score <0.1 OR MaxEnt <5% and SSFL <15%.

**BP5:**

- **Supporting**: a further (likely) pathogenic variant was observed in the proband.

BP6: does not apply

**BP7**:

- **Strong**: Splice variants where RNA studies have confirmed no impact.

- **Supporting**: A synonymous (silent) variant for which splicing prediction algorithms predict no impact to the splice consensus sequence nor the creation of a new splice site AND the nucleotide is not highly conserved.

**TESE procedure**

All patients recruited in Doha underwent microTESE, whereas in Barcelona, microTESE was reserved for patients with elevated FSH (>20 IU/L) and low testicular volume (<10 ml). The remaining patients, as well as all patients from Newcastle and Nijmegen, underwent conventional TESE. Briefly, conventional TESE was performed under local anesthesia, involving a small scrotal incision and the excision of testicular tissue samples from multiple locations in both testes. In contrast, microTESE was conducted under general anesthesia. The testicle was delivered through a scrotal incision, followed by a lateral and equatorial incision in the tunica albuginea to expose the seminiferous tubules. The testicular tissue was examined under an operating microscope, and the larger, more opaque seminiferous tubules were selectively excised. The same procedure was then repeated for the contralateral testicle. If no dilated tubules were identified, multiple random biopsies were taken from all testicular compartments.

**REFERENCES**

Abou Tayoun AN, Pesaran T, DiStefano MT, Oza A, Rehm HL, Biesecker LG, Harrison SM, ClinGen Sequence Variant Interpretation Working Group (ClinGen SVI). Recommendations for interpreting the loss of function PVS1 ACMG/AMP variant criterion. *Hum Mutat* 2018;**39**:1517–1524.

Biesecker LG, Harrison SM, ClinGen Sequence Variant Interpretation Working Group. The ACMG/AMP reputable source criteria for the interpretation of sequence variants. *Genet Med* 2018;**20**:1687–1688.

Brnich SE, Abou Tayoun AN, Couch FJ, Cutting GR, Greenblatt MS, Heinen CD, Kanavy DM, Luo X, McNulty SM, Starita LM, *et al.* Recommendations for application of the functional evidence PS3/BS3 criterion using the ACMG/AMP sequence variant interpretation framework. *Genome Med* 2019;**12**:3.

Chen X, Schulz-Trieglaff O, Shaw R, Barnes B, Schlesinger F, Källberg M, Cox AJ, Kruglyak S, Saunders CT. Manta: rapid detection of structural variants and indels for germline and cancer sequencing applications. *Bioinformatics* 2016;**32**:1220–1222.

Geoffroy V, Lamouche J-B, Guignard T, Nicaise S, Kress A, Scheidecker S, Le Béchec A, Muller J. The AnnotSV webserver in 2023: updated visualization and ranking. *Nucleic Acids Res* 2023;**51**:W39–W45.

Harrison SM, Biesecker LG, Rehm HL. Overview of Specifications to the ACMG/AMP Variant Interpretation Guidelines. *Curr Protoc Hum Genet* 2019;**103**:e93.

Jarvik GP, Browning BL. Consideration of Cosegregation in the Pathogenicity Classification of Genomic Variants. *Am J Hum Genet* 2016;**98**:1077–1081.

Krausz C, Riera-Escamilla A, Moreno-Mendoza D, Holleman K, Cioppi F, Algaba F, Pybus M, Friedrich C, Wyrwoll MJ, Casamonti E, *et al.* Genetic dissection of spermatogenic arrest through exome analysis: clinical  implications for the management of azoospermic men. *Genet Med* 2020;**22**:1956–1966.

Li H, Durbin R. Fast and accurate short read alignment with Burrows-Wheeler transform. *Bioinformatics* 2009;**25**:1754–1760.

McLaren W, Gil L, Hunt SE, Riat HS, Ritchie GRS, Thormann A, Flicek P, Cunningham F. The Ensembl Variant Effect Predictor. *Genome Biol* 2016;**17**:122.

Oud MS, Smits RM, Smith HE, Mastrorosa FK, Holt GS, Houston BJ, Vries PF de, Alobaidi BKS, Batty LE, Ismail H, *et al.* A de novo paradigm for male infertility. *Nat Commun* 2022;**13**:154. England.

Poplin R, Ruano-Rubio V, DePristo MA, Fennell TJ, Carneiro MO, Auwera GA Van der, Kling DE, Gauthier LD, Levy-Moonshine A, Roazen D, *et al.* Scaling accurate genetic variant discovery to tens of thousands of samples. 2017;

Rausch T, Zichner T, Schlattl A, Stütz AM, Benes V, Korbel JO. DELLY: structural variant discovery by integrated paired-end and split-read analysis. *Bioinformatics* 2012;**28**:i333–i339.

Wyrwoll MJ, Köckerling N, Vockel M, Dicke A-K, Rotte N, Pohl E, Emich J, Wöste M, Ruckert C, Wabschke R, *et al.* Genetic Architecture of Azoospermia-Time to Advance the Standard of Care. *Eur Urol* 2023;**83**:452–462.


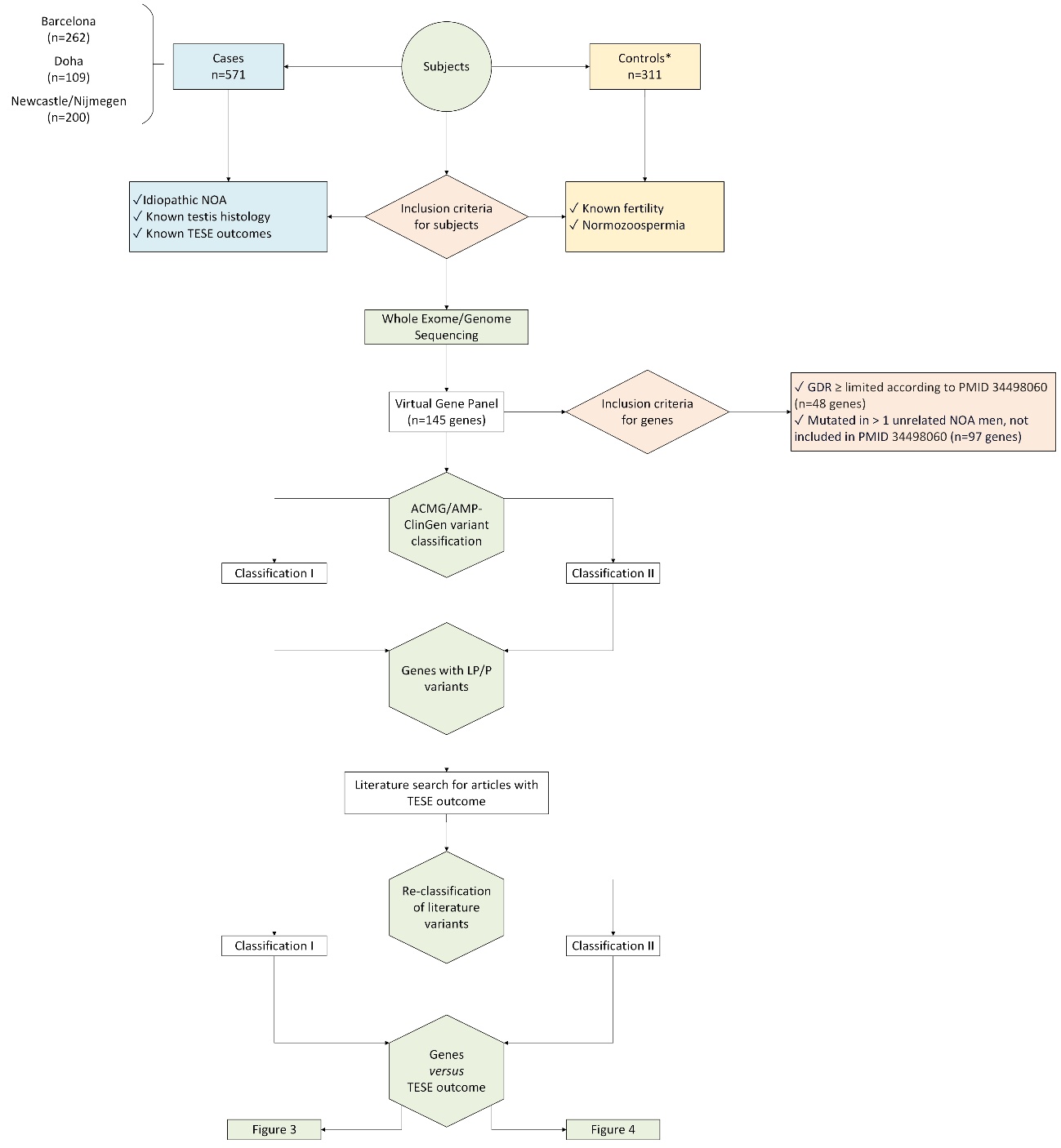


**Supplementary Figure S1.** Schematic representation of the study design

Abbreviations: NOA, Non-Obstructive Azoospermia; TESE, Testicular Sperm Extraction; ACMG, American College of Medical Genetics and Genomics; AMP, Association for Molecular Pathology; GDR, Gene-Disease Relationship; LP, Likely Pathogenic; P, Pathogenic. * Control individuals were enrolled in Barcelona.

**Panel A Panel B**


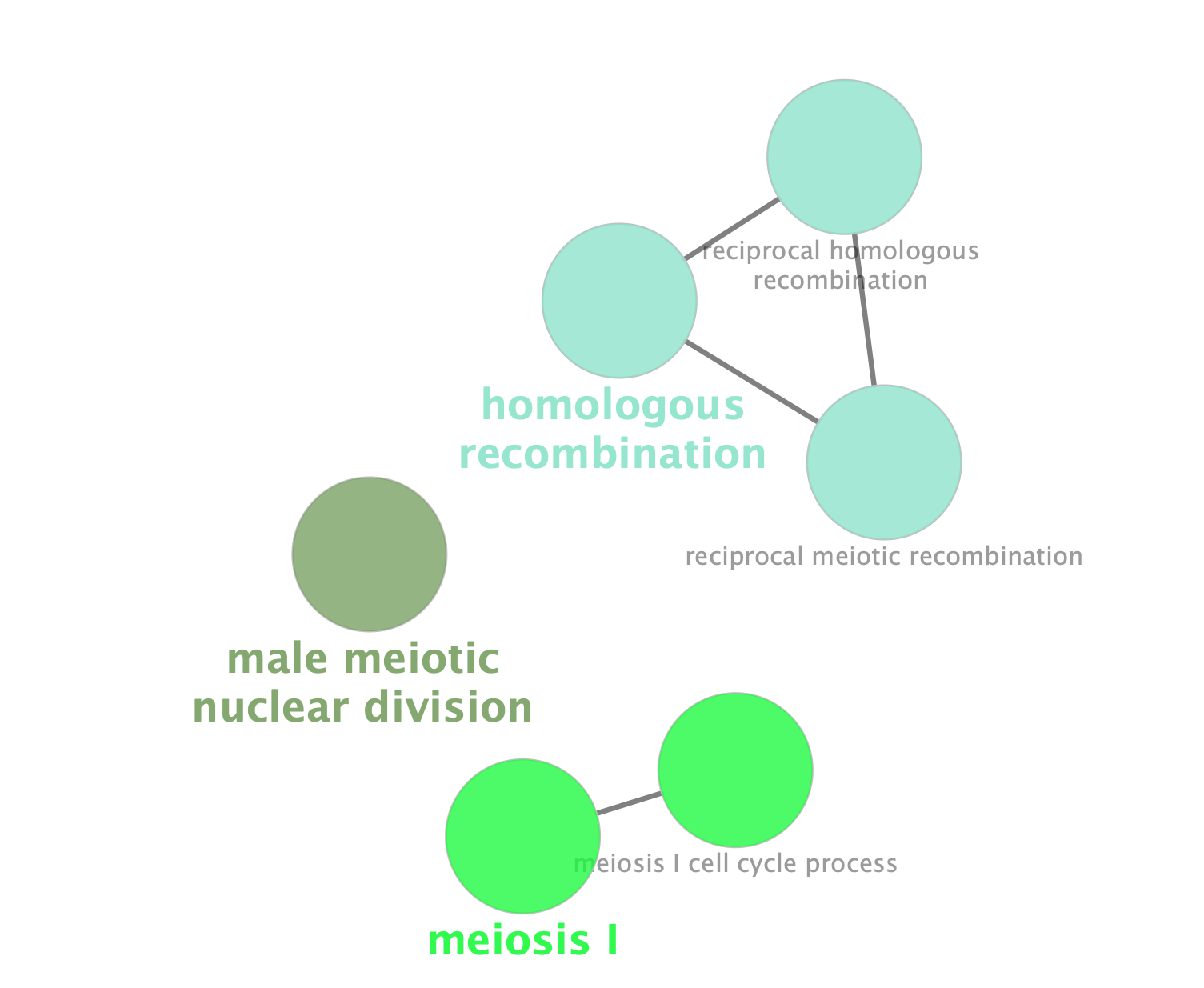
**
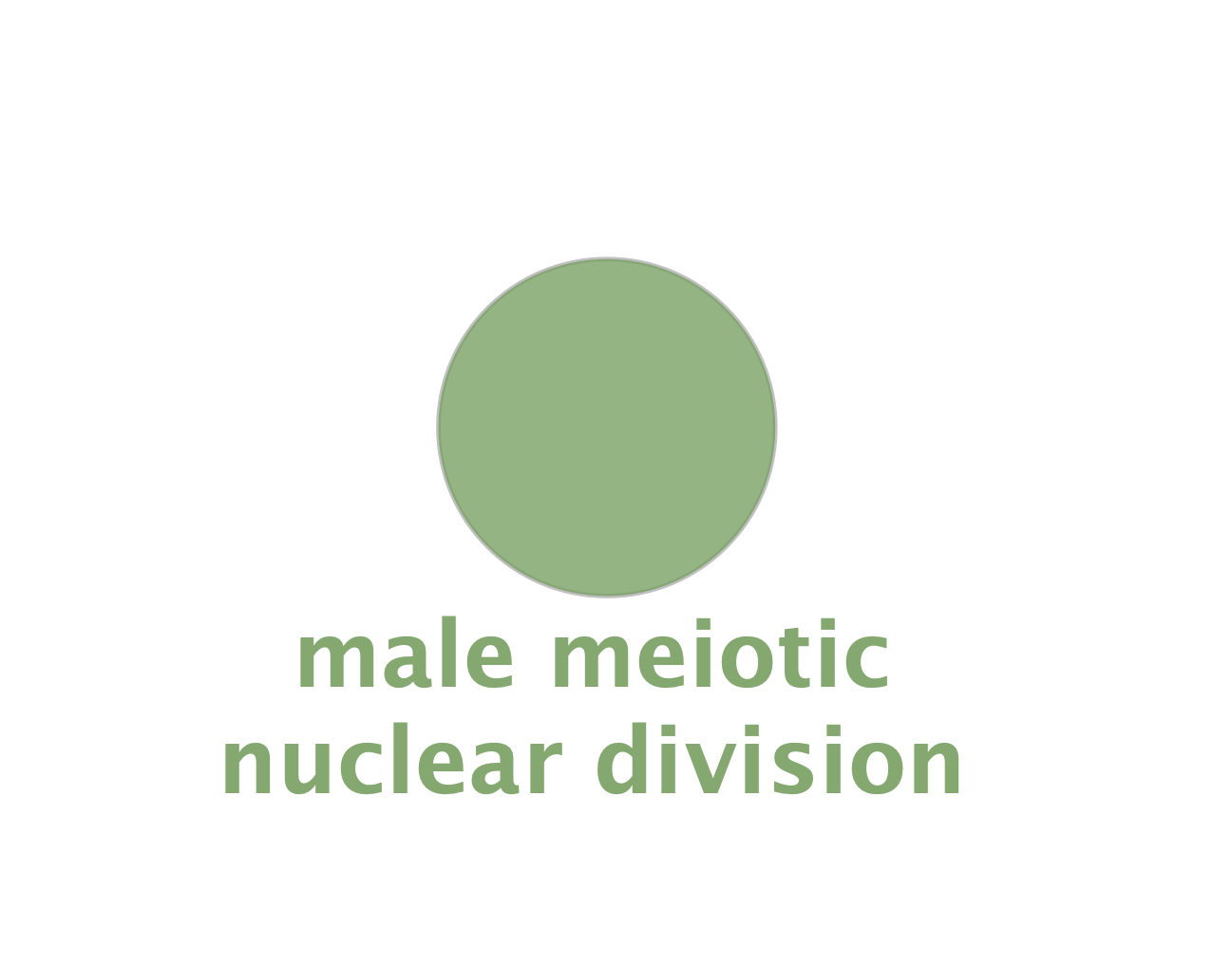
**

**Supplementary Figure S2.** Network of biological processes affected by genes with LP/P variants identified using the ACMG/AMP adapted from Wyrwoll et al. (2023) (Panel A) and ACMG/AMP-based variant interpretation framework refined to genetic diagnostics in non-obstructive azoospermia (Panel B) linked to positive TESE outcomes. Redundant GO terms are collapsed into common biological themes. Each node represents a term affected by at least three genes in the disease-linked gene set.

**Panel A Panel B**


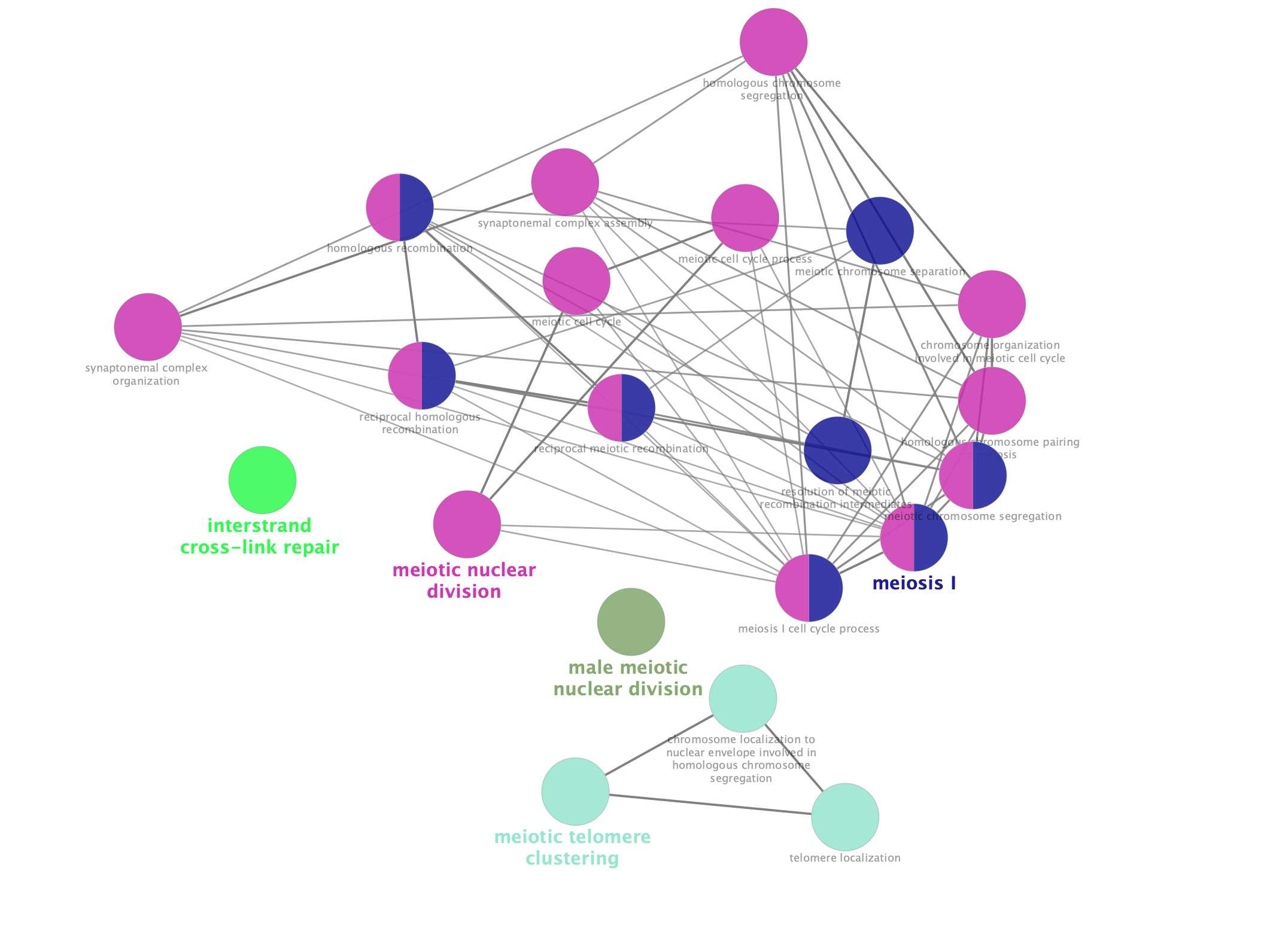

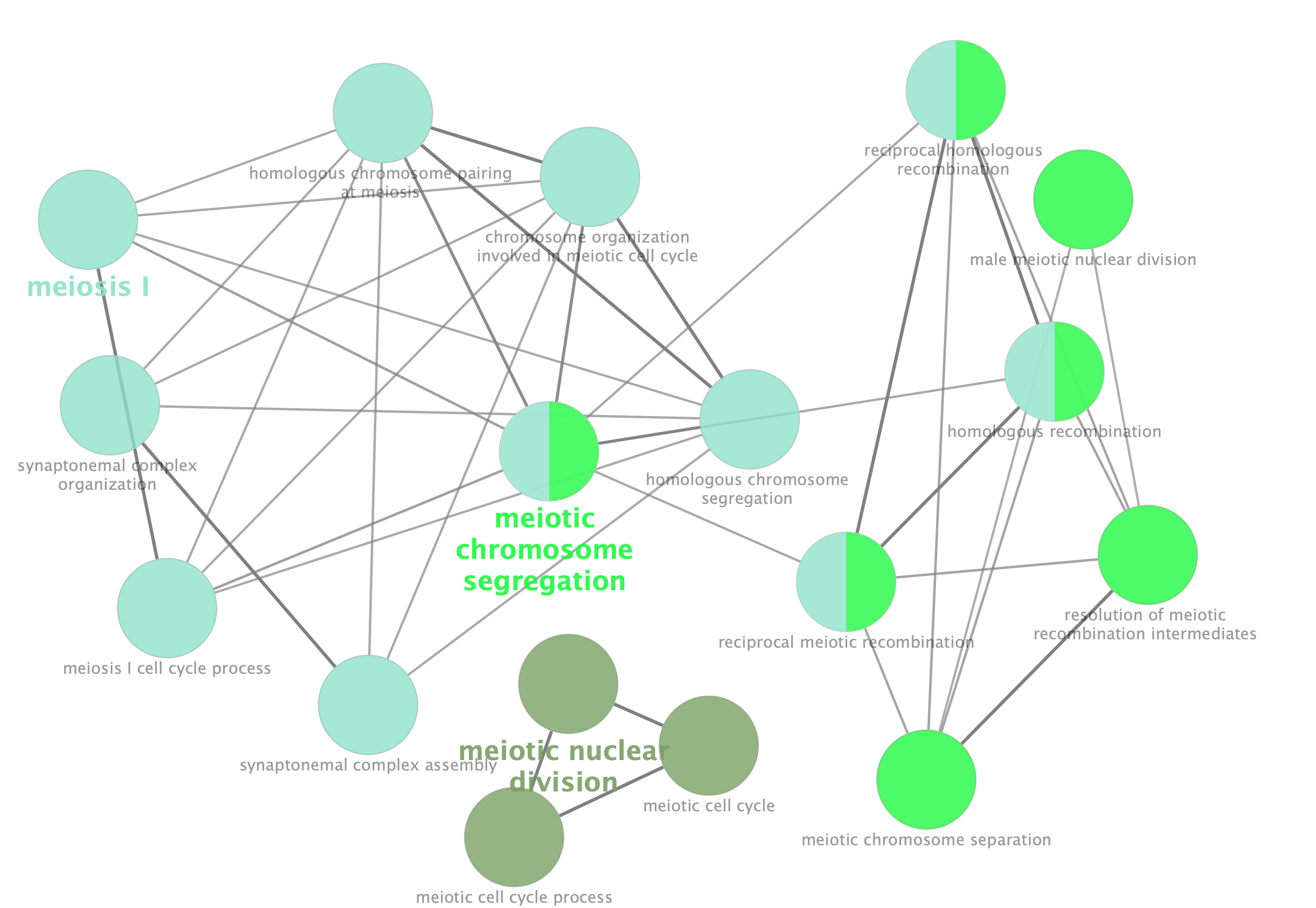


**Supplementary Figure S3.** Network of biological processes affected by genes with LP/P variants identified using the ACMG/AMP adapted from Wyrwoll et al. (2023) (Panel A) and ACMG/AMP-based variant interpretation framework refined to genetic diagnostics in non-obstructive azoospermia (Panel B) linked to negative TESE outcomes. Redundant GO terms are collapsed into common biological themes. Each node represents a term affected by at least three genes in the disease-linked gene set.

**Supplementary Table S1: Summary of the sperm parameters from the normozoospermic control cohort.**

| Sperm parameter | Median [interquartile] |
| --- | --- |
| Sperm concentration (M/ml) | 111 [78-161.25] |
| Total Sperm Count (M/ejaculate) | 378.5 [252-553.58] |
| Progressive motility (%) | 55.5 [47-64] |
| Morphology (% of typical forms) | 13 [8-21] |

**Supplementary Table S5: Summary of the comparisons performed on carriers of VUS and LB/B variants according to two different ACMG-AMP variant classification methods. Comparisons were performed in function of TESE outcome.**

| **A)** | **Carriers of variants according to ACMG classification and method used**  **n (%)** | | | |
| --- | --- | --- | --- | --- |
| **TESE outcome** | **VUS** | | **LB/B** | |
| ***Entire cohort*** (n=571) | **Variant classification I** (n=200) | **Variant classification II**  (n=114) | **Variant classification I** (n=8) | **Variant classification II**  (n=123) |
| *Negative* (n=329, 57.6%) | 104 (31.42) | 72 (21.75) | 2 (0.6) | 66 (19.94) |
| *Positive* (n=242, 42.4%) | 96 (39.34) | 42 (17.21) | 6 (2.46) | 57 (23.36) |
| Two sided  *p value*, OR (CI 95%) | 0.62, 0.71 (0.50-1.00) | **0.01***, 1.34 (0.88-2.04) | 0.29, 0.24 (0.05-1.21) | 0.47, 0.82 (0.55-1.22) |
| ***BCN cohort*** |  |  |  |  |
| *Negative* (n=142) | 47 (33.1) | 34 (23.94) | 2 (1.41) | 31 (21.83) |
| *Positive* (n=120) | 58 (48.33) | 20 (16.67) | 6 (5) | 31 (25.83) |
| ***Doha cohort*** |  |  |  |  |
| *Negative* (n=71) | 31 (43.66) | 21 (29.58) | 0 (0) | 19 (26.76) |
| *Positive* (n=38) | 17 (44.74) | 11 (28.95) | 0 (0) | 9 (26.68) |
| ***NCL/NIJ cohort*** |  |  |  |  |
| *Negative* (n=116) | 26 (22.03) | 17 (14.41) | 0 (0) | 16 (13.56) |
| *Positive* (n=84) | 21 (24.42) | 11 (12.79) | 0 (0) | 17 (19.78) |
|  | | | | |

Statistical significance is indicated by asterisks as follows: p < 0.05; ** p < 0.01; *** p < 0.001. Statically significant results are highlighted in bold..

Abbreviations: ACMG, American College of Medical Genetics; AMP: Association for Molecular Pathology; Variant classification I: ACMG/AMP-based, adapted from Wyrwol et al 2023; Variant classification II: ACMG/AMP-based variant interpretation framework refined to genetic diagnostics in non-obstructive azoospermia; VUS, variant of uncertain significance; LB/B, likely benign/benign; TESE, Testicular Sperm Extraction; OR, odds ratio; CI, confidence interval; BCN, Barcelona; NCL/NIJ, Newcastle/Nijmegen.
