# Supplementary material for: Genetic determinants of testicular sperm extraction outcomes: insights from a large multicentre study of men with non-obstructive azoospermia
Source: Hum Reprod Open. 2025 Aug 29;2025(3):hoaf049. doi: 10.1093/hropen/hoaf049 (PMC12396851; doi:10.1093/hropen/hoaf049)
Supplement: hoaf049_Supplementary_Data [file hoaf049_supplementary_data.zip › HRO-25-0045-R2-Table1.docx]

**Table 1. Summary of the comparisons performed on carriers of LP/P variants according to two different ACMG-AMP variant classification methods; comparisons were performed in function of TESE outcome or testis phenotype.**

|  |  | **Carriers of LP/P variants according to different classification methods**  **n (%)** | |
| --- | --- | --- | --- |
|  | **Entire cohort** (n=571) | **Variant Classification I** (n=64) | **Variant Classification II** (n=35) |
| *TESE outcome* | Negative (n=329, 57.6%) | 53 (16.11) | 30 (9.11) |
|  | Positive (n=242, 42.4%) | 11 (4.51) | 5 (2.06) |
|  | Negative versus positive  p value, OR (CI 95%) | **6.03x10^-8***^**, 4.13 (2.11-8.08) | **1.29x10^-5***^**, 4.99 (1.89-12.9) |
| *Testis phenotype* | MA (n=171) | 34 (19.9) | 20 (11.7) |
|  | SCO (n=238) | 22 (9. 2) | 11 (4.6) |
|  | HSG (n=143) | 8 (5.6) | 4 (2.8) |
|  | MA versus SCO  p value, OR (IC 95%) | **0.0158***, 2.31 (1.26-4.29) | 0.0680, 2.48 (1.12-5.75) |
|  | MA versus HSG  p value, OR (IC 95%) | **0.0011****, 4.2 (1.83-10.89) | **0.0173***, 4.62 (1.5-19.03) |

Odds ratios (OR) with 95% confidence intervals (CI) were calculated using the Wald method. The binomial exact test was used to compare variant frequencies between TESE-positive and TESE-negative groups under a two-sided hypothesis. Differences in the frequency of likely pathogenic/pathogenic (LP/P) variants among testicular phenotypes (SCO, MA, HSG) were assessed using Chi-square tests. P-values from pairwise comparisons (SCO vs. MA, SCO vs. HSG, MA vs. HSG) were adjusted for multiple testing by the Bonferroni method. Statistical significance is indicated by asterisks as follows:p < 0.05; ** p < 0.01; *** p < 0.001. Statistically significant results are highlighted in **bold**.

Abbreviations: ACMG, American College of Medical Genetics; AMP, Association for Molecular Pathology; Variant classification I: ACMG/AMP-based, adapted from Wyrwoll et al. 2023; Variant classification II: ACMG/AMP-based variant interpretation framework refined to genetic diagnostics in non-obstructive azoospermia; LP/P, likely pathogenic/pathogenic; VUS, variant of uncertain significance; LB/B, likely benign/benign; TESE, Testicular Sperm Extraction; OR, odds ratio; CI, confidence interval; SCO, Sertoli cell-only; MA, maturation arrest; HSG, hypospermatogenesis.
